# Physiological and Proteomic Analysis of the Rice Mutant *cpm2* Suggests a Negative Regulatory Role of Jasmonic Acid in Drought Tolerance

**DOI:** 10.3389/fpls.2017.01903

**Published:** 2017-11-10

**Authors:** Rohit Dhakarey, Manish L. Raorane, Achim Treumann, Preshobha K. Peethambaran, Rachel R. Schendel, Vaidurya P. Sahi, Bettina Hause, Mirko Bunzel, Amelia Henry, Ajay Kohli, Michael Riemann

**Affiliations:** ^1^Molecular Cell Biology, Institute of Botany, Karlsruhe Institute of Technology, Karlsruhe, Germany; ^2^International Rice Research Institute, Los Baños, Philippines; ^3^Newcastle University Protein and Proteome Analysis, Newcastle University, Newcastle Upon Tyne, United Kingdom; ^4^Department of Food Chemistry and Phytochemistry, Institute of Applied Biosciences, Karlsruhe Institute of Technology, Karlsruhe, Germany; ^5^Cell and Metabolic Biology, Leibniz Institute of Plant Biochemistry, Halle, Germany

**Keywords:** jasmonates, rice, drought, root, proteomics, phytohormones, cross-talk

## Abstract

It is widely known that numerous adaptive responses of drought-stressed plants are stimulated by chemical messengers known as phytohormones. Jasmonic acid (JA) is one such phytohormone. But there are very few reports revealing its direct implication in drought related responses or its cross-talk with other phytohormones. In this study, we compared the morpho-physiological traits and the root proteome of a wild type (WT) rice plant with its JA biosynthesis mutant *coleoptile photomorphogenesis 2* (*cpm2)*, disrupted in the allene oxide cyclase (AOC) gene, for insights into the role of JA under drought. The mutant had higher stomatal conductance, higher water use efficiency and higher shoot ABA levels under severe drought as compared to the WT. Notably, roots of *cpm2* were better developed compared to the WT under both, control and drought stress conditions. Root proteome was analyzed using the Tandem Mass Tag strategy to better understand this difference at the molecular level. Expectedly, AOC was unique but notably highly abundant under drought in the WT. Identification of other differentially abundant proteins (DAPs) suggested increased energy metabolism (i.e., increased mobilization of resources) and reactive oxygen species scavenging in *cpm2* under drought. Additionally, various proteins involved in secondary metabolism, cell growth and cell wall synthesis were also more abundant in *cpm2* roots. Proteome-guided transcript, metabolite, and histological analyses provided further insights into the favorable adaptations and responses, most likely orchestrated by the lack of JA, in the *cpm2* roots. Our results in *cpm2* are discussed in the light of JA crosstalk to other phytohormones. These results together pave the path for understanding the precise role of JA during drought stress in rice.

## Introduction

Rice serves as the staple food for more than 50% of world's population. It is cultivated in over a hundred countries, with a total harvested area of ~163.3 million hectares, with a produce of more than 749.7 million tons annually (GRiSP, 2013; FAO, 2016). In comparison to other crops, rice production is highly water-intensive and almost 30.9% of the total rice area of the world is through rainfed agriculture (Wassmann et al., 2009; Dixit et al., 2014). As per an estimate, nearly 2,500 liters of water is needed to give rise to 1 kg of rice, and irrigated rice consumes around 34–43% of the world's total irrigation water (Bouman et al., 2007). Hence water deficit or drought is said to be the most calamitous form of abiotic stress for rice and can result in yield losses of 15-50%, depending on the severity and timing of stress (Srividhya et al., 2011). Globally, drought stress leads to an estimated 18 million tons of rice yield reduction per year (O'Toole, 2004).

Drought stress affects rice during all growth stages (Venuprasad et al., 2008). Development of drought-tolerant, higher-yielding varieties suitable for water-limiting environments is a major challenge for improvement of rice production and in turn for increasing food security. The development of such varieties can be fast-tracked through the knowledge of the physiological and molecular genetic mechanisms underpinning drought tolerance.

Growth regulators also known as phytohormones orchestrate plant responses to growth, development, and external stimuli (Wolters and Jurgens, 2009). These compounds are synthesized through various biosynthetic processes and can function either at the site of their genesis or be translocated to function somewhere else in the plant. They often quickly modify gene expression by instigating or blocking the degradation of transcriptional regulators via the ubiquitin—proteasome system (Santner and Estelle, 2010). Abscisic acid (ABA)-mediated signaling and ABA-responsive genes is one of the most investigated hormonal responses of plants to abiotic stress, especially in drought (Tuteja, 2007; Sreenivasulu et al., 2012). The role of ABA in the regulation of stomata opening/closure and other critical responses to abiotic stress is quite established (Mittler and Blumwald, 2015). Drought and other stresses such as, salinity result in an increased content of ABA, which orchestrates extensive changes in the expression of genes regulated by or responsive to ABA (Shinozaki and Yamaguchi-Shinozaki, 2007). However, such a change in a single phytohormonal pathway usually affects other phytohormonal pathways as well, and the resulting crosstalk contributes to the overall response of plant to abiotic stress (Kohli et al., 2013). Jasmonates (i.e., jasmonic acid, its precursors, and direct derivatives) are critical components of such crosstalk (Riemann et al., 2015).

The phytohormone jasmonic acid (JA) and its metabolically active derivatives like methyl-jasmonate and JA-Isoleucine (JA-Ile) are crucial signaling molecules, which actively engage in plant responses to various biotic as well as abiotic stresses (Wasternack, 2007; Balbi and Devoto, 2008). In the JA biosynthesis pathway, plastidial 13-lipoxygenases (13-LOX) act on linolenic acid to convert it to 13*S*-hydroperoxyoctadecatrienoic acid (13-HPOT). Allene oxide synthase (13-AOS) and allene oxide cyclase (AOC) act in combination to synthesize *cis*-12-oxophytodienoic acid (OPDA). OPDA is then transported into peroxisomes where it is reduced by OPDA reductase (OPR). Subsequently it is converted into JA by three β-oxidation steps (Wasternack and Hause, 2013). An important step in jasmonate biosynthesis is regulated by AOC, which is encoded by a four membered small gene family in *Arabidopsis thaliana*. In *A. thaliana*, AOC functions as homo- or hetero-trimer (Hofmann et al., 2006; Stenzel et al., 2012; Otto et al., 2016). In rice, however, this enzyme is encoded by a single copy gene (Dhakarey et al., 2016).

There is limited information on the role of jasmonates in the response of rice to drought (Liu et al., 2015; Riemann et al., 2015; Dhakarey et al., 2016). In this report, a JA synthesis mutant called *coleoptile photomorphogenesis 2 (cpm2)* was used to understand the role of JA on rice physiological and molecular responses to drought. It carries an 11 bp deletion within the first exon of *AOC* (Riemann et al., 2013; Dhakarey et al., 2016). Comparative studies between the WT and the mutant for differences in drought response were undertaken at the vegetative growth stage, since *cpm2* is male sterile. Assessment of drought response was conducted on a set of morphological parameters and root proteome. The differentially abundant proteins (DAPs) in the root proteome study were related to various pathways and processes. Additional histological and metabolite analysis guided by the DAPs supported the observation that *cpm2* was better adapted to cope with drought stress. This leads to the proposition that jasmonates might be negative regulators of drought tolerance.

## Materials and methods

### Plant growth and drought stress conditions for phenotyping and stomatal conductance

*Oryza sativa* L. *ssp. japonica* cv. Nihonmasari serving as the wild type (WT) and the *cpm2*, the JA biosynthesis mutant generated in the same background (Riemann et al., 2013), were used in this whole study. Dehusked caryopses were surface sterilized as per Hazman et al. (2015), were then sown on 0.5% phytoagar medium (Duchefa, Netherlands) and kept for 14 days in a culture room at 25°C and continuous light of 120 μmol quanta m^−2^s^−1^. On the 14th day, 10 selected seedlings of each genotype were transferred in sand filled pots, kept for another 2 weeks under short-day conditions of 10 h light at 28°C and 280 μmol quanta m^−2^s^−1^, followed by 14 h darkness at 22°C in a phytochamber (BBC York, Mannheim, Germany). Plants were kept well-watered and provided once a week with fertilizers “Wuxal-TopN” (N 141.6 g/l, P_2_O_5_ 47.2 g/l, K_2_O 70.8 g/l, B 0.118 g/l, Cu 0.047 g/l, Fe 0.236 g/l, Mn 0.141 g/l, Mo 0.012 g/l, Zn 0.047 g/l) and “Wuxal-Super” (N 99.2 g/l, P_2_O_5_ 99.2 g/l, K_2_O 74.4 g/l, B 0.124 g/l, Cu 0.049 g/l, Fe 0.248 g/l, Mn 0.148 g/l, Mo 0.012 g/l, Zn 0.049 g/l (Manna, Ammerbuch-Pfaffingen, Germany). In the phytochamber, five seedlings of each genotype (aged 28 days) were subjected to drought stress for 4 days by withholding water followed by rewatering for 2 days; control plants were maintained in parallel. Stomatal conductance was measured with a portable-type porometer (Decagon Devices, USA) for 4 consecutive days after withholding water and for 2 consecutive days after rewatering at noon on the youngest fully-expanded attached leaf on the plants of the same age (five sub-replicates).

### Root architecture and water use efficiency (WUE) analysis setup

Root architecture and WUE experiments were conducted in the greenhouse at the IRRI. For the root architecture study, seedlings germinated as described above were planted in small (40 cm height, 4 cm in diameter, 502.6 cm3) cylinders filled with dry, sterilized and sieved soil to a bulk density of 1.1 g cm^−3^. Soil moisture treatments of control (100% of field capacity), 75% of field capacity and dry-down to 50% of field capacity were applied as described by Henry et al. (2015). Five replicates of each genotype were planted for each soil moisture treatment. Plants were scouted regularly to guard against insect or pathogen infestations; sticky fly paper traps were also suspended between the cylinders to keep away insects. Roots were harvested, subjected to washing in order to remove the dirt and soil prior to their storage in 75% ethanol for further analysis using WinRHIZO (Régent Instruments, Quebec, Canada).

Similarly, for apparent WUE measurement, WT, and *cpm2* seedlings were planted in large cylinders (95 cm in height, 20 cm in diameter, 29.8 l) which were filled with dried soil previously brought from an upland IRRI farm and were arranged in randomized complete block design. Additional sterilized soil was filled at the top of each cylinder. Ten plants of each genotype were planted in 20 large cylinders placed inside a concrete tank (1.35 m deep, 3.5 m wide, and 6.8 m in length) inside the naturally ventilated greenhouse. All cylinders were equally watered twice a day to keep them watered but not flooded. Complete fertilizer was also applied as per Kijoji et al. (2012) 1 day after transplanting Drought stress (DS) was imposed on five plants of each genotype at 32 days after sowing by draining water from the designated cylinders and by withholding further addition of water in them. The top of the cylinders were then covered with polythene sheets to curtail direct evaporation, so that only water loss by transpiration was measured. Remaining cylinders belonging to the control treatment were kept watered using the target weight to maintain the soil moisture level at field capacity, which was established by weighing at day 36 after sowing. DS cylinders were not watered for the remaining time. Digital imaging of shoots and weighing of each cylinder was performed three times per week using the system described by Kijoji et al. (2012) for 4 weeks after imposing drought stress. Using *image J* software (NIH, USA) with an integrated macro language, leaf area was estimated from digital images and divided by the water uptake rate to calculate apparent WUE.

### Plant materials, growth, and stress conditions for root proteome, ABA, cell wall metabolite, and transcript level analysis

Sampling material for experiments related to root proteome, transcript and selected metabolite analysis were raised in the naturally ventilated greenhouses at IRRI during the 2014 and 2015 dry season. WT and *cpm2* seedlings generated as described above were then transferred to plastic pots (20 cm in diameter) filled with autoclaved soil (previously collected from IRRI upland farm). All pots were equally watered by providing 200 ml twice a day for 1 week and then separated into “control” and “stressed” categories. For the control treatment, plants were watered but not flooded, and their entire roots and shoots were sampled at day 22. For drought-stressed plants, watering was stopped to initiate moderate and severe stress condition. Volumetric soil moisture content (SMC) in the drought-stress treatment was monitored by ML3 Theta Probe soil moisture sensor (Delta-T Devices, London, UK; one per replicate). The entire root and shoot was sampled for “stressed” treatments as soon as SMC reached 30 and 15% for moderate and severe stress, respectively, and were instantly frozen in liquid nitrogen. Soil particles were removed from the root samples prior to their storage in liquid nitrogen.

### Protein extraction, separation, tryptic digestion, and TMT labeling

Frozen root samples from WT and *cpm2* were used for total protein extraction. Subsequently, a modified trichloroacetic acid (TCA, Merck, USA) method was used for protein extraction. Frozen root samples (~250 mg) were crushed to a fine powder in a pre-cooled mortar with liquid nitrogen. Afterwards, the powder was transferred to a 1.5 ml pre-chilled Eppendorf tube and then following extraction procedure was performed: (i) 700 μl of freshly prepared 10% TCA in acetone with 0.07% DTT (DL-Dithiothreitol, Merck, USA) was added to the tubes. The tubes were then placed at −80°C for 1 h, and then centrifuged in a pre-cooled rotor at 13,000 g for 10 min at 4°C (Beckman Allegra 64R preparative centrifuge, USA). (ii) The supernatant was discarded and the pellet was re-suspended in 700 μl of freshly prepared pre-cooled 10% acetone with 0.07% DTT. The suspension was further stored at −80°C for 30 min, prior to centrifugation in a pre-cooled rotor at 13,000 g for 10 min at 4°C. This step was repeated twice. The supernatant was discarded and the pellet was then air-dried for 45 min in a laminar hood at room temperature (~24°C). The resulting pellet was then re-suspended in 700 μl solubilizing buffer containing 9 M urea, 1% DTT, 4% CHAPS (3-[(3-Cholamidopropyl) dimethylammonio]-1-propane sulfonate), 35 mM Tris base, 5 μM EDTA (Ethylenediaminetetraacetic acid), 1 μM PMSF (phenylmethylsulfonyl fluoride) and 10 μl of protease inhibitor cocktail solution (Merck, USA). Afterwards, the samples were centrifuged at 13,000 g for 5 min at room temperature and the resulting supernatant with the proteins was collected into a clean Eppendorf tube. The proteins were run through SDS-PAGE under denaturing conditions (Laemmli, 1970). The subsequent in-gel digestions of proteins, TMT labeling and Nano-LC-MS/MS analysis were performed as previously published (Raorane et al., 2015a). For overview, refer to Figure S1.

### Protein identification, functional annotation, and evaluation

Protein identification and their functional annotation was performed as described by Raorane et al. (2015a). For the proteins common to both WT and *cpm2* roots, a threshold of log ratio 1.0 or greater (2-fold more) was used as a criterion for comparative abundance of the protein in one genotype. Conversely, proteins having log ratios lower than 1.0 (2-fold less) were considered comparatively less abundant.

### Quantitative reverse transcriptase PCR (qRT-PCR) analysis of selected genes

Primer3 was used for designing the primers (listed in Table S1) and the subsequent procedures related to RNA extraction; cDNA synthesis and qRT-PCR were performed as illustrated in Raorane et al. (2015b). *OsCyclophilin-2* was employed as the housekeeping gene for all the qRT-PCR analysis (Pabuayon et al., 2016).

### Quantification of endogenous ABA and jasmonate levels in shoot and root samples

ABA and jasmonates were quantified from shoot and root samples as described by Balcke et al. (2012). In brief, about 50 mg of frozen plant material was homogenized and extracted with 500 μl pure methanol supplied with [^2^H_6_] ABA, [^2^H_5_] OPDA, [^2^H_6_] JA, and [^2^H_2_] JA-Ile (50 ng each) as internal standards. After centrifugation, the supernatant was diluted with 9 volumes of water and subjected to solid phase extraction on HR-XC (Chromabond, Macherey-Nagel) column. Elution was done with 900 μl acetonitrile. Ten micro liter of the eluate was subjected to ultra-performance liquid chromatography–tandem mass spectrometry (UPLC-MS/MS) according to Balcke et al. (2012). The contents of ABA, OPDA, JA, and JA-Ile were calculated using the ratio of analyte and internal standard peak heights.

### Root cell wall monosaccharides and hydroxycinnamic acids analysis

Sampling material grown at the IRRI greenhouses (for targeted metabolite analysis) was used for these measurements. Further milling of the frozen root samples was done to <0.5 mm (IKA mill or porcelain mortar with dry ice). Milled samples were stored in the freezer (−44°C) for subsequent monosaccharides and hydroxycinnamic acids analyses. Destarched cell wall material was isolated and purified (digestion with thermostable α-amylase and amyloglucosidase and precipitation of non-starch polysaccharides in 80% ethanol). Ester-linked oligo-ferulates (dimers and trimers) were quantified in the destarched cell wall material following alkaline hydrolysis (2 M NaOH, 18 h), acidification, extraction with diethyl ether, and LC-ESI-MS/MS separation and detection (negative ionization with triple-quadrupole mass analyzer). Internal standard quantification was performed using 5–5-methyl-diferulic acid. Ester-linked monomeric *trans-*ferulic and *trans-p*-coumaric acids (FA and *p*CA respectively) were also determined in the alkaline hydrolysates, but were detected and quantified via external curves using an inline UV detector (320 and 307 nm detection wave lengths for *trans*-ferulic and *trans-p*-coumaric acids respectively) coupled to the LC-MS/MS system. Monosaccharide analysis of the destarched cell wall material was performed after sulfuric acid hydrolysis using high-performance anion-exchange chromatography with pulsed amperometric detection (HPAEC-PAD) as described in Schäfer et al. (2016).

### Histology of root tissue

WT and *cpm2* seedlings were grown in a phytochamber (BBC York, Mannheim, Germany) as described above. Root samples were harvested for control and severe stress (SMC 15%) treatments. Using a hand microtome, thin sections of 25–30 μm thickness were taken from the center of basal and axial ends of roots from WT and *cpm2* plants grown normally and under drought stress conditions. Sections were stained in 1% (w/v) phloroglucinol in 18% hydrochloric acid (HCl), mounted with water on a glass slide and observed using an Axioskop microscope (Zeiss GmbH, Jena, Germany) equipped with a 20x objective. Pictures were taken using a CCD camera (AxioCam, Zeiss) attached to the microscope.

### Statistical analysis

Representation of individual plants of a genotype within each sample and the number of replicates for each analysis/measurement are given in the figure legends. Student's *t*-test was performed for testing the statistical significance of the calculated mean values, with a significance level of *P* ≤ 0.05 and subsequent graphs were plotted using GraphPad Prism version 7 (GraphPad Software, La Jolla California USA). The ASReml script was used as a repeated measures analysis across all dates for WUE using R v. 3.3.1 (R Core Team, 2016). The underlying statistical model was WUE~Treatment + Genotype + DAS.

## Results

### Increased adaptive response of *cpm2* morpho-physiology to drought

*cpm2* and WT plants raised for 4 weeks were subjected to drought stress. After withholding water for 2 days, response to drought became apparent in the WT plants through leaf rolling, mainly in the upper reaches of the leaves, despite apparently stronger culms, compared to *cpm2* (Figures S2A,B). At the end of day four, WT leaves developed symptoms of wilting. In contrast the upper reaches of the *cpm2* leaves remained open and appeared turgescent while still the well-developed culm of the WT gave the impression of a stronger plant (Figures S2C,D). These initial results obtained with plants in the phytochamber were further confirmed by greenhouse studies in tropical latitudes.

**Figure 1:**
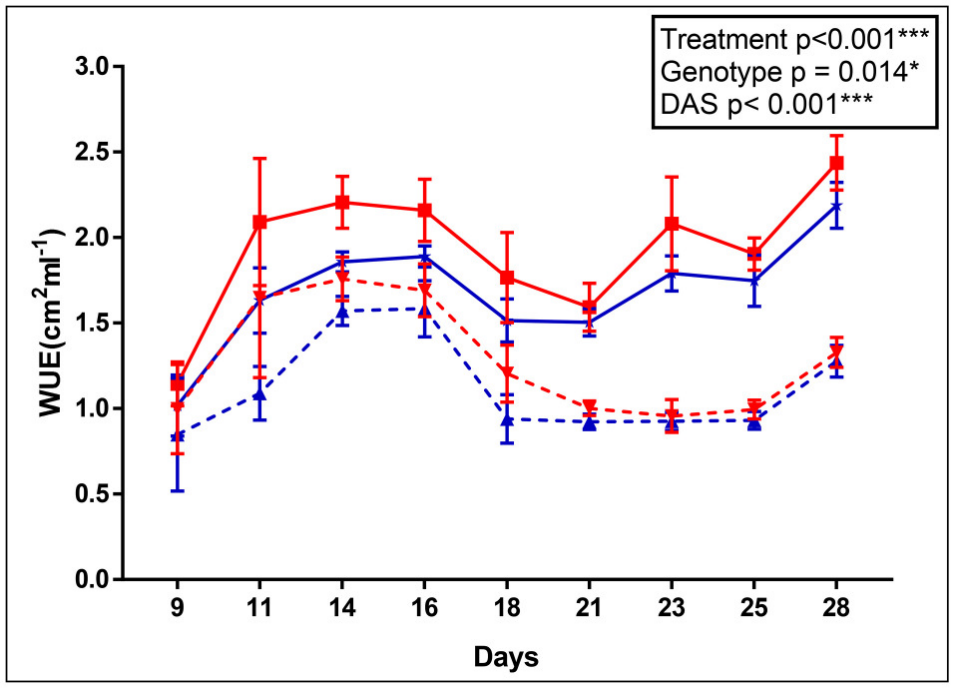
Apparent water use efficiency (WUE) of wild type (WT) and *cpm2*. WT& *cpm2* plants were subjected to drought after 32 days of sowing by withholding water. Digital imaging of shoots was performed to estimate green leaf area which was later normalized by water uptake/day to calculate apparent WUE. Data is shown for 9, 11, 14, 16, 18, 21, 23, 25 and 28 days after imposing drought stress. The values presented are means ± standard error(SE); *n* = 5. WT and *cpm2* are indicated by blue and red lines, respectively; whereas dotted and solid lines represent control and stress treatment, respectively. The *p*-value is indicated as determined by ASReml analysis. Significance levels are indicated as : ^*^P ≤ 0.05; ^***^P < 0.001.

The apparent adaptation of *cpm2* to drought was borne out by other parameters. For example, the *cpm2* plants had a higher water use efficiency (WUE) before drought stress was initiated as compared to WT (0.23 unit higher in *cpm2* in ASReml). However, with progressive drought stress, WUE in *cpm2* plants was even higher than in those of WT (0.28 units higher in *cpm2* in ASReml) and the difference increased persistently till day 14 after the onset of drought (Figure 1). Consistently, the *p*-value for the genotype effect became lower under drought conditions as well.

**Figure 2:**
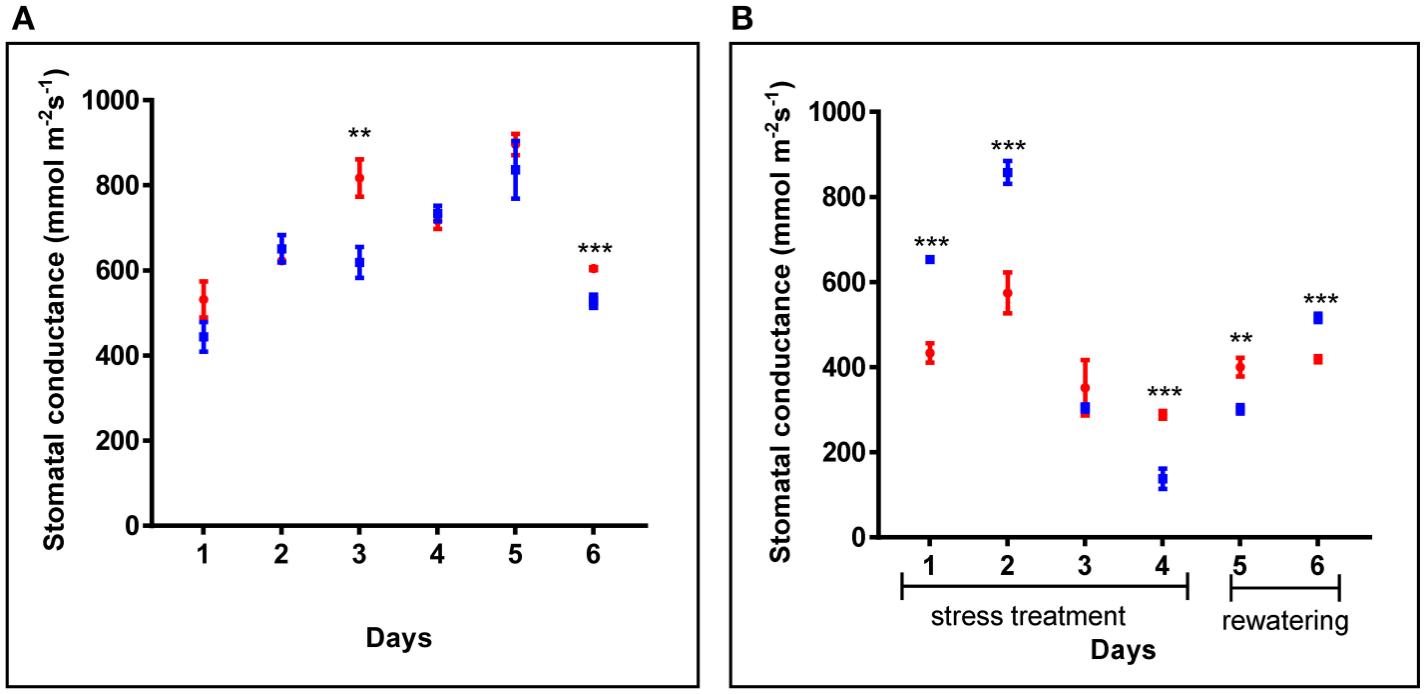
Comparison of stomatal conductance of wild type (WT) and *cpm2* in **(A)** control conditions and **(B)** under drought. Four-week-old WT and *cpm2* plants were exposed to drought stress by withholding water for 4 days followed by re-watering for 2 days. Under control conditions, WT and *cpm2* maintained almost a steady state throughout the course of experiment, while *cpm2* maintained lower stomatal conductance as compared to WT under drought. The values presented are means ± standard error (SE); *n* = 5. Blue and red symbols indicate WT and *cpm2*, respectively. Stars (^**^ and ^***^) denote statistical significance (*P* ≤ 0.01, and *P* ≤ 0.001), respectively, between the two genotypes in a Student's *t*-test.

Additionally, in 4 weeks old plants, similar stomatal conductance was observed for *cpm2* and WT plants under control conditions (Figure 2A). However, under drought condition, the overall stomatal conductance in *cpm2* showed less drastic decrease than in the WT (Figure 2B). Initially, during the first couple of days after the onset of drought conditions, stomatal conductance in WT showed significant increase compared to *cpm2*. However, at day three, the stomatal conductance for both the genotypes decreased significantly, however both maintained almost similar degree of conductance. From day four, the trend was reversed and stomatal conductance in *cpm2* plants was 2-fold more than in WT plants (Figure 2B), which were almost wilting (Figure S2C). After re-watering, water levels in both sets of plants were replenished. One day after re-watering, *cpm2* plants still exhibited higher stomatal conductance compared to the WT plants. However, stomatal conductance progressively increased in the WT plants after re-watering, till on day 6 it was more than that in the *cpm2* plants (Figure 2B).

**Figure 3:**
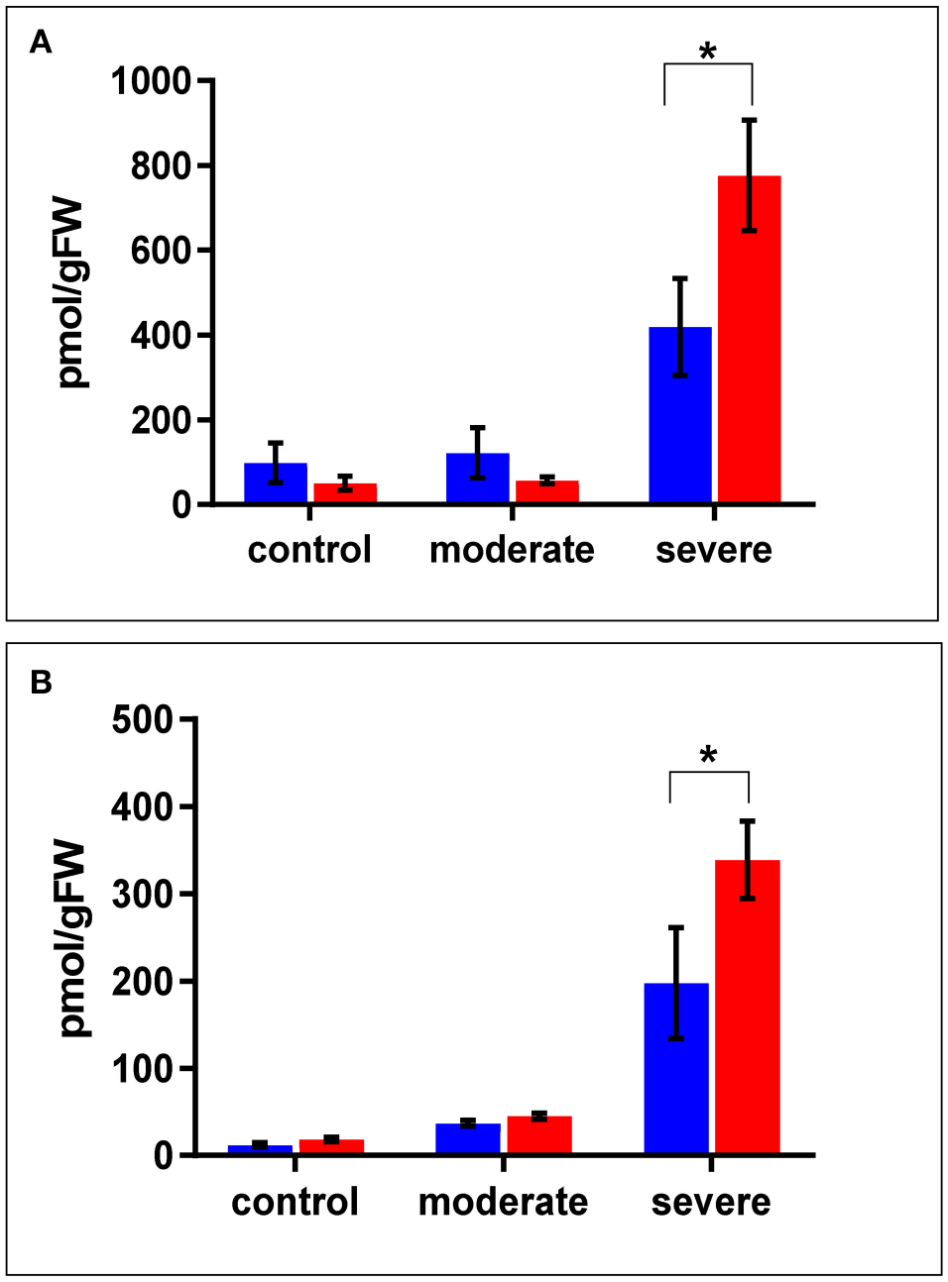
The level of ABA (Abscisic Acid) in **(A)** shoots and **(B)** roots of *cpm2* and wild type (WT) under control, moderate, and severe drought stress. Three-week-old WT and *cpm2* seedlings were either kept well-watered, and the control plants were sampled at day22, or watering was stopped to initiate moderate and severe stress conditions. Shoot and root samples were harvested in control, moderate and severe stress condition, respectively. WT and *cpm2* are represented by blue and red bars, respectively. Data represents mean value ± standard error (SE); *n* = 5. Star (^*^) denote statistical significance (*P* ≤ 0.05), respectively, between the two genotypes in a Student's *t*-test.

In terms of drought responsive phytohormones, the ABA content is a measure of drought tolerance (Daszkowska-Golec, 2016; Negin and Moshelion, 2016). Under both the control and moderate stress conditions (SMC of 30%), ABA content in the WT shoots was almost double that in *cpm2* shoots. Under severe stress conditions (SMC of 15%), ABA increased in both *cpm2* and WT shoots. However, the increase in ABA content in *cpm2* shoots was almost double that in the WT shoots (Figure 3A). In case of root ABA content, there were no significant changes in their levels in both the genotypes under control and moderate stress, however, during severe stress *cpm2* had almost 1.5 fold induction in the ABA content as compared to the WT (Figure 3B).

As assessed through leaf rolling, WUE, stomatal conductance, and ABA content, these results clearly demonstrated *cpm2* to have an advantage over WT in coping with drought stress. An advantageous drought response of the aerial parts suggested that the roots of *cpm2* may also exhibit advantageous features as roots first sense and react to drought.

### The root system of *cpm2* is better developed

**Figure 4:**
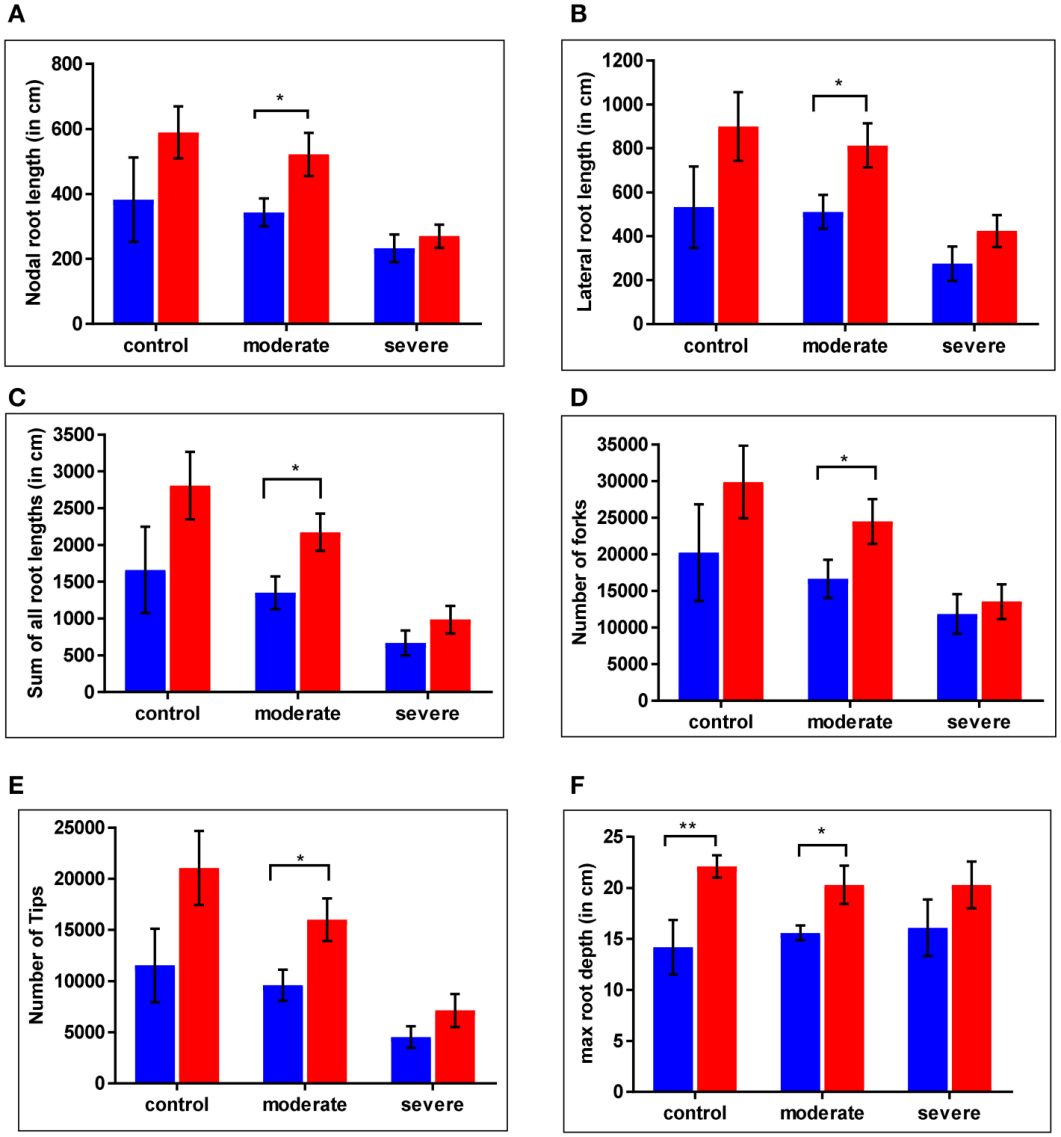
Comparative differences were observed in root architecture in wild type (WT) and *cpm2*. Plants of two genotypes were grown in soils at different moisture levels. Roots were harvested, washed and stored in 75% ethanol until scanning and analysis. Significant differences were observed across the treatments. Values for **(A)** nodal root length **(B)** lateral root length **(C)** sum of all root length **(D)** number of forks **(E)** number of tips **(F)** max root depth are shown here. Data represents mean value ± standard error (SE); *n* = 5. WT and *cpm2* are represented by blue and red bars, respectively. Stars (^*^ and ^**^) denote statistical significance (*P* ≤ 0.05 and *P* ≤ 0.01), respectively, between the two genotypes in a Student's *t*-test.

For the WT and *cpm2* plants root growth was assessed under control and drought stress conditions. Nodal root length was reduced under severe drought by nearly 40% in the WT and by nearly 50% in *cpm2* compared to the control condition (Figure 4A). Lateral root length was reduced under drought in both plant types by nearly 50% compared to control condition, whereby severe drought stress inflicted more drastic reduction than moderate stress (Figure 4B). Thus, *cpm2* did not exhibit a comparative advantage in nodal and lateral root length under severe drought. A similar profile was observed for other root traits such as, the sum of all root lengths (Figure 4C) and the total number of root forks and tips (Figures 4D,E). However, in all these aforementioned root traits a significant advantage was observed in *cpm2* plants under control conditions and this advantage was further maintained during moderate stress conditions compared to WT. Also, in *cpm2*, maximum root depth (Mrd) exceeded that of the WT in all the treatments (Figure 4F). However, this difference in Mrd between WT and *cpm2* became smaller with increasing severity of drought stress. Under severe drought stress the difference was no longer statistically significant. These results highlighted that overall *cpm2* exhibited a more branched root system than the WT both under control and drought conditions (Figure S3).

### Root proteome analysis supports *cpm2* adaptation to drought

**Figure 5:**
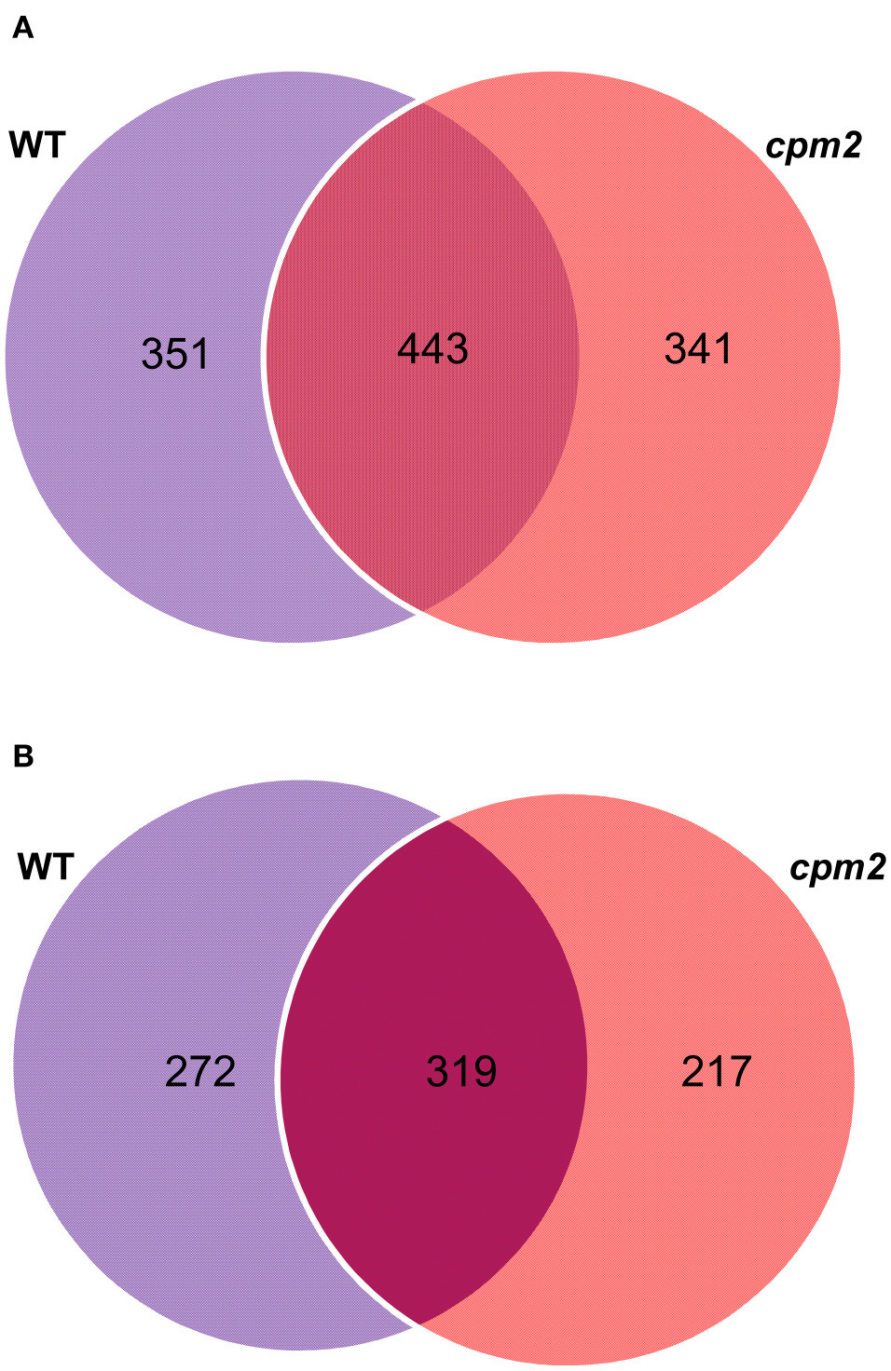
Venn diagrams of proteins obtained after TMT (Tandem Mass Tag) analysis. 4,194 hits were assigned to unique peptides leading to combined identification of a total of 1,578 proteins in wild type (WT) and *cpm2* roots. **(A)** 351 and 341 proteins were uniquely identified in WT and *cpm2* roots, respectively, while 443 proteins were common to both. **(B)** Number of proteins quantified were 272 and 217 unique ones in WT and *cpm2*, respectively, with 319 common proteins.

Root peptide extracts from *cpm2* and WT plants under control and severe drought were subjected to mass spectrometry analysis using high-throughput Tandem Mass Tag (TMT) approach. Out of 15,172 spectra obtained in total, 13,485 referred 4,573 peptides. Of these, 4,194 hits were designated to unique peptides, which amounted to the combined identification of a total of 1,578 proteins in WT and *cpm2* roots. Out of these, 351 and 341 proteins were unique to WT and *cpm2* roots, respectively, whereas 443 proteins were common in both the genotypes (Figure 5A). However, the number of unique proteins with dependable quantitative estimates was 272 and 217 for WT and *cpm2*, respectively, and 319 as common proteins (Figure 5B). In order to filter out JA-dependent and –independent proteins, a comparison between control and drought conditions was carried out within one genotype (WT or *cpm2*, respectively).

A wide range of isoelectric points (4.41–11.91) and molecular weights (4.4–236.7 kDa) implicated a dynamic range of proteins. Proteins from both the genotypes were assigned to 32 functional categories using MapMan (Thimm et al., 2004) as per the rice mapping file (Figure S4).

**Figure 6:**
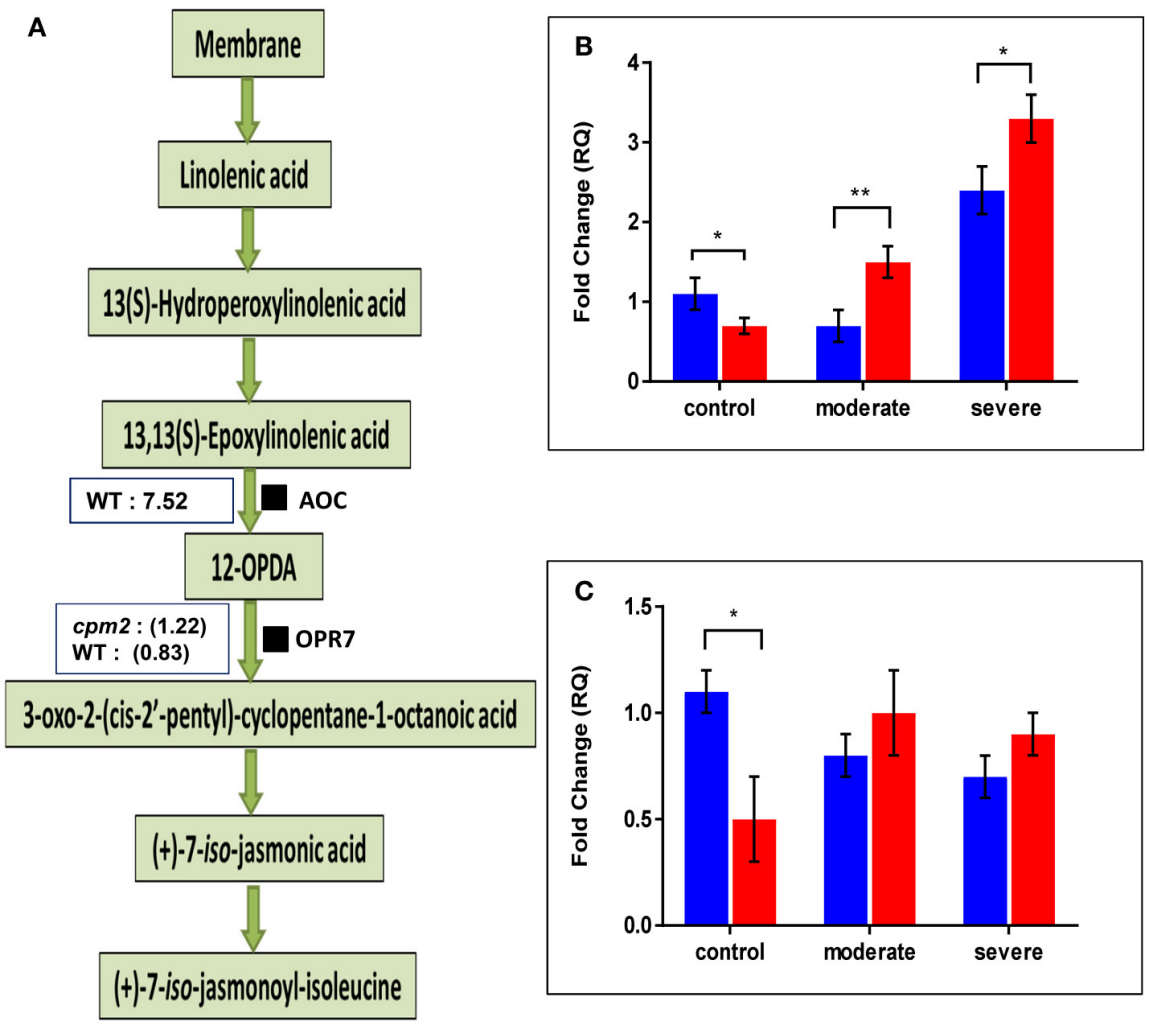
Effect of drought on enzymes involved in JA biosynthesis and their transcript levels. **(A)** Overview of the jasmonic acid pathway with log ratios (stress/control) for AOC and OPR7protein are shown in boxes, respectively. Transcript abundances of mRNAs encoding **(B)** AOC and **(C)** OPR7protein were analyzed at control, moderate and severe drought stress conditions. Wild type (WT) and *cpm2* are represented by blue and red bars, respectively. Data represents mean value ± standard error (SE); *n* = 3. Stars (^*^ and ^**^), denote statistical significance (*P* ≤ 0.01 and *P* ≤ 0.001), respectively, between the two genotypes in a Student's *t*-test.

The AOC protein was highly abundant in WT but as expected, it was not found in *cpm2* (Figure 6A). Lack of AOC protein was nevertheless not directly related to its transcript abundance. In fact, under moderate or severe drought, *AOC* transcript was abundant in *cpm2* as compared to the WT (Figure 6B). Under well-watered condition, the WT contained nearly 2-fold more *AOC* transcript than *cpm2*. The *AOC* transcripts in *cpm2* are thus either not translated, or the protein may have been highly unstable, and the mutational frame-shift in the coding sequence leading to an undesirable protein may account for that. In contrast to AOC, the oxo-phytodienoate reductase (OPR7), an enzyme operating downstream of AOC (Tani et al., 2007), was more abundant in *cpm2* under drought (Figure 6A). The *OPR7* transcript levels were also observed to be significantly higher in WT than in *cpm2*. However, under moderate and severe drought stress no significant difference in the expression of *OPR7* was observed (Figure 6C).

Apart from such differences owing to the mutation, many proteins showed substantial up- or down-regulation (difference of log ratio of 1.0) and were classified as DAPs between the WT and the mutant plant. Several of such proteins could be assigned to categories responsive to drought. These categories, the genes therein and their protein abundance status in the WT and *cpm2* are summarized in Table 1. The potential importance of such DAPs in the apparent adaptation of *cpm2* to drought is described in the “Discussion” section.

**Table 1:** List of Differentially abundant Proteins (DAPs) showing highest abundance in WT and *cpm2* under drought stress.

**S. No**	**Category**	**Protein**	Amount^*^		**Function**
			***cpm2***	**WT**	
**1**	**ROS SCAVENGING**				
		Glutathione S-transferase (OsGSTF2)			Antioxidant
		Glutathione S-transferase (OsGSTU12)			Antioxidant
		Ascorbate peroxidase (OsAPX7),			Antioxidant
		DJ-1 family protein (DJ-1/Pfp)			Methyl-glyoxal detox & antioxidant
		Serine hydroxymethyltransferase (SHMT)			Serine-glycine-glutathione antioxidant
		3-isopropylmalate dehydratase small subunit			Leucine biosynthesis and antioxidant
		Non-symbiotic hemoglobin 2 (nsHb2)			Oxygen sensor
**2**	**CELL WALL**				
		glycosyl hydrolase family 17 (Gns6)			Modify cell wall polysaccharides
		UDP-glucose-6-dehydrogenase (UDGH)			Nucleotide sugars for cell wall
		O-methyltransferase (OMT)			Lignin biosynthesis
		Polygalacturonase (PG)			Pectin degradation
**3**	**CYTOSKELETON**				
		β-Tubulin 1			Microtubules
		Actin nucleation protein			Microfilaments
**4**	**PHENYLPROPANOIDS**				
		Phenylalanine-ammonia-lyase (OsPAL1)			Antioxidative & lignin biosynthesis
		4-coumarate-CoA ligase (Os4CL3)			“
		Caffein-CoA-methyltransferase (OsCCoAOMT)			”
		Cinnamyl alcohol dehydrogenase (OsCAD4)			“
		Caffein-o-methyltransferase (OsCOMT1)			”
**5**	**ENERGY**				
		Triose phosphate isomerase (TPI)			Glycolysis
		Pyruvate decarboxylase (PDC)			Ethanolic fermentation
		Glyceraldehyde 3-phosphate dehydrogenase (GAPDH)			Glycolysis
**6**	**PROTEIN CATABOLISM**				
		Peptidase T1 family			Protein peptidase
		Peptidase T1 family			Protein peptidase
**7**	**MISCELLANEOUS**				
		Aquaporin (OsPIP1-2)			Water channel
		Ras related protein (OsRAS1)			G Protein signaling cascade
		Glutamine synthase (OsGS1)			Nitrogen remobilization

* Abundance of protein is shown by contrasting shades. White color in column refers to absence of protein. See Figure 8 for details

### Cell wall metabolite analysis supports root phenotype and proteome-based inferences

**Figure 7:**
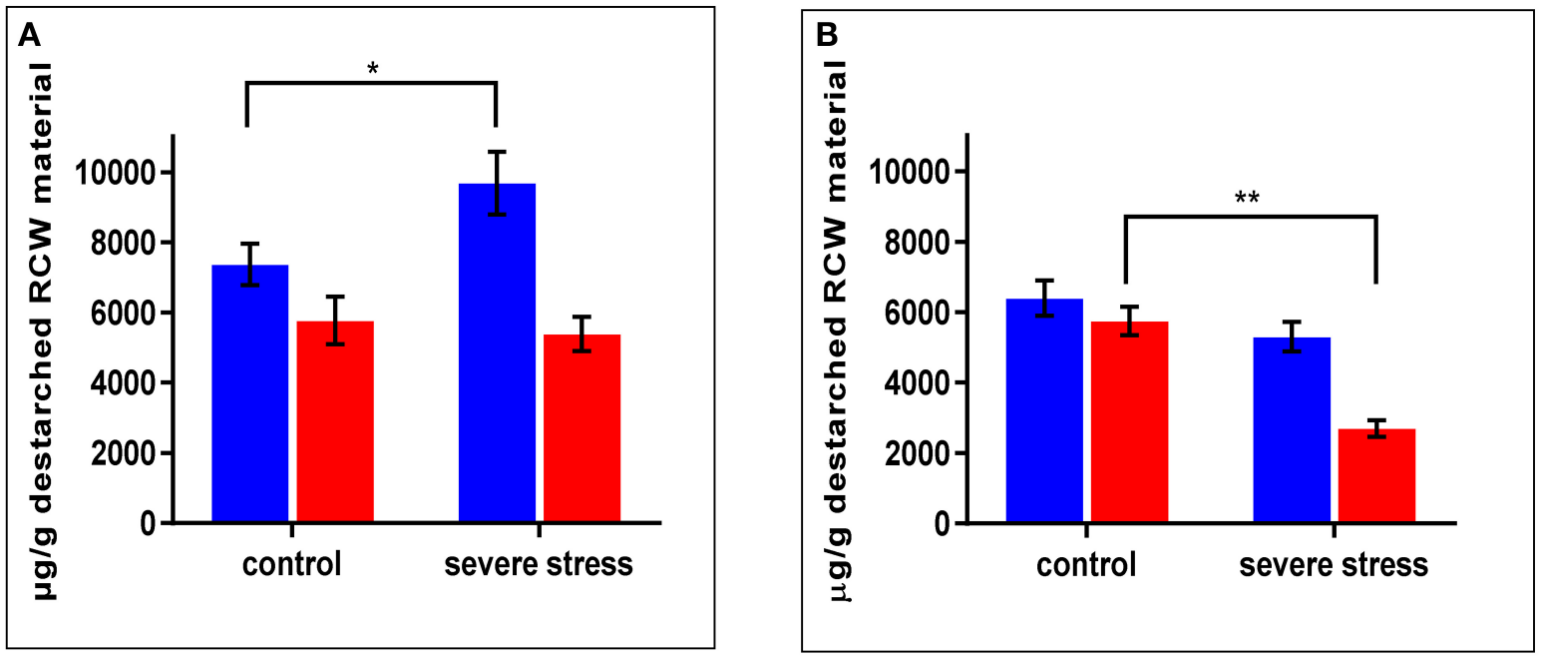
Ester linked **(A)** Coumaric Acid) **(B)** Ferulic Acid levels in wild type (WT) and *cpm2* destarched root cell wall (RCW) material under control and severed rought stress conditions. Both the metabolites in the cell wall-bound state were less in roots exposed to drought in *cpm2*. Three-week-old WT and *cpm2* seedlings were either kept well-watered, and their roots were sampled at day 22, or watering was stopped to initiate severe stress condition. Root samples were harvested in control and severe stress condition. WT and *cpm2* are represented by blue and red bars, respectively. Data represents mean value ± standard error (SE); *n* = 5. Stars (^*^ and ^**^) denote statistical significance (*P* ≤ 0.05 and *P* ≤ 0.01), respectively, between the two genotypes in a Student's *t*-test.

As mentioned above, *cpm2* root traits such as more branching, increased total root length and root depth exhibited a genotypic advantage which was maintained under stress. This was supported by root proteome-based information, mainly on the basis of DAPs that can potentially modify or affect the cell wall structure, composition or properties (Table 1). In *cpm2* there was an increased content of six major enzymes of the phenylpropanoid pathway involving cinnamic acid modification, which potentially modify the cell wall composition. The enzymes involved suggest variation in coumaric and ferulic acid content (Figure S5), which was confirmed by the quantification of the coumaric and ferulic acid ester-linked to cell wall biopolymers in the roots. Ester-linked coumaric and ferulic acid contents were both significantly lower in *cpm2* compared to the WT under drought (Figures 7A,B). This agreed with the increased content of the enzymes that used the two acids as substrates in *cpm2*. The levels of ester-linked di-, tri- and total oligo-ferulates were also generally lower in the roots of *cpm2* under drought but significantly so for the measured triferulates (Figure S6C). Under control conditions, there was no significant difference between WT and *cpm2* in the content of coumaric or ferulic acid or ferulate oligomers (Figure 7, Figure S6).

## Discussion

### Higher WUE and stomatal conductance in *cpm2* under drought stress

The observation of higher WUE and higher stomatal conductance in *cpm2* is somewhat antagonistic. Genotypes bearing higher stomatal conductance may indicate a pronounced capability of uptaking available soil water through enhanced root area (Mitchell et al., 1996) or osmotic adjustment (Blum, 2005), and thereby, can maintain transpiration during moderate drought stress (Blum, 2009). But on the other hand, enhanced stomatal conductance might be a downside when soil water deficits are more common (Donovan et al., 2007). Plants counter severe water deficit by decreasing their stomatal conductance, however by a curtailment of WUE, plants could deplete available water quickly and consequently die by loss of turgor, if they fail in a drought survival strategy (Donovan et al., 2007; Manzoni et al., 2011). In the case of our study, the higher stomatal conductance may be an effect of greater root length and branching in *cpm2*. Increase in ABA content under drought is known to be related to stomatal closure in leaves, which increases WUE (Gowing et al., 1990; Zhang and Davies, 1990; Gomes et al., 1997). In fact, the different accumulation of ABA in the shoots of the WT and *cpm2* are in agreement with the differences observed in their stomatal conductance (Figures 2, 3A). One could anticipate that due to the high ABA concentration in *cpm2* stomatal closure could even be more pronounced as we could observe in our measurements. One explanation for this could be that ABA may need interaction with JA to stimulate stomatal closure (Daszkowska-Golec and Szarejko, 2013). Since *cpm2* lacks JA, the stomata may not have been affected to the degree expected despite the increase in ABA. One possibility for a limited effect of ABA under the lack of JA could be the fact that JA upregulates ABA receptor genes (Lackman et al., 2011), which could thus be limiting in *cpm2*.

Despite the antagonistic features, *cpm2* survived severe drought stress better than the WT. Higher stomatal conductance as well as higher WUE under drought was noted before in some genotypes of peanuts, where root length density was positively correlated to WUE (Songsri et al., 2013). Limiting vegetative growth also increases WUE. The *cpm2* plants showed reduced culm development (Figure S2D), which is also supported by lower shoot dry weight under drought in *cpm2* (Figure S7). Overall, through the aerial morpho-physiological parameters of leaf rolling, WUE, stomatal conductance, and ABA content as well as the root architecture of increased branching indicated that *cpm2* plants had a better potential to tolerate drought.

### Root proteome-based insights into drought tolerance by *cpm2*

A more branched root system as in the case of *cpm2* (Figure 4, Figure S3) may provide a significant advantage to counter drought as seen by cross-validating a near isogenic line (NIL) and a transgenic line in field samples of a large-effect QTL for yield under drought (Dixit et al., 2015; Raorane et al., 2015a). During drought stress, the root is the primary organ to sense water scarcity in drying soil, triggering a cascade of responses at morpho-physiological, biochemical, and cellular levels (Sengupta et al., 2011). Hence comparative proteomic studies on roots were undertaken. Adaptive growth of the *cpm2* roots under drought was evidently supported through various DAP categories.

#### DAPs in ROS scavenging enzymes are more abundant in *cpm2*

**Figure 8:**
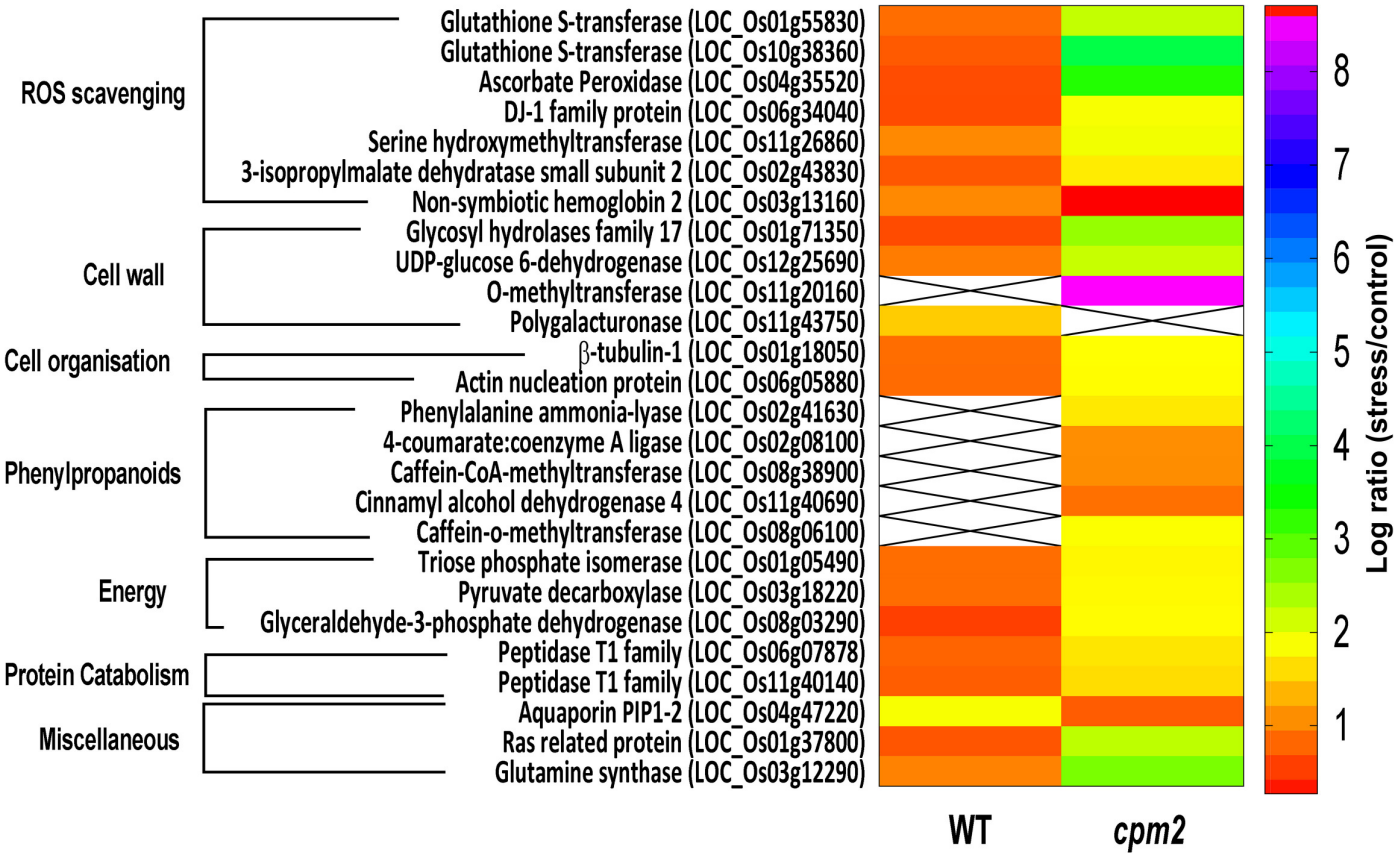
Heat map of differentially abundant proteins (DAPs) belonging to ROS scavenging, cell wall, cell organization, phenylpropanoids, energy, proteincatabolism and miscellaneous categories obtained after TMT (Tandem Mass Tag) analysis of *cpm2* and wild type (WT) roots. Differential proteins were abundant in *cpm2* (except nSHB2 and PIP-2) as evident from the log ratios (stress/control). TMT based LC-MS/MS analysis was performed on rice roots after drought treatment of *cpm2* and corresponding WT. Total protein was extracted and subsequent FASP digestion was performed. The subsequent peptide mixture was labeled with the TMT reagent prior to multiplexing. Pooled peptides were fractioned using the reversed-phase HPLC system and then individual fractions were analyzed using Tandem mass spectrometry. For each protein the ID according to MSU is provided in parenthesis. The color scale indicates whether a protein is more or less abundant under drought conditions. Cross marks indicate that the protein was unidentified in the respective sample.

Two glutathione-S-transferases (GST; OsGSTF2, and OsGSTU12) were more abundant in *cpm2* (Figure 8). GSTs are well known for their role in detoxification of xenobiotics, they also work as antioxidants by tagging and removal of oxidative degradation products, especially from fatty acids and nucleic acids (Moons, 2005). GSTs may also act as glutathione peroxidase to directly scavenge peroxides (Frova, 2003). Also, ascorbate peroxidase (APX) participates in ascorbate-glutathione cycle, and hence is functional in free radical detoxification (Cramer et al., 2013). In *cpm2*, the OsAPX7 was also more abundant (Figure 8). Both glutathione (GSH) and ascorbate (AsA) are also important non-enzymatic antioxidants for plant defense against oxidative stress (Foyer and Noctor, 2005). In *cpm2*, some other proteins with antioxidant activities were also more abundant. Under various abiotic and biotic conditions in plants, serine hydroxymethyltransferases reportedly participate in dissipative mechanisms to curtail the production of ROS (Moreno et al., 2005). Their antioxidant activity may also be related to the serine-glycine-glutathione synthesis pathway. One serine hydroxymethyltransferase, OsSHMT2, was more abundant in *cpm2* (Figure 8). Similarly, a DJ-1 family protein was also more abundant in *cpm2* (Figure 8). They are known to possess antioxidant property reportedly through detoxifying glyoxal/methyl-glyoxal (Ghosh et al., 2016), and accelerated cell-death is also reported due to their loss of function (Xu et al., 2010). A 3-isopropylmalate dehydrogenase (3-IPMD) was also more abundant in *cpm2* (Figure 8). This enzyme is involved in leucine and glucosinolate biosynthesis (He et al., 2009). Glucosinolates act by inducing antioxidative enzymes such as GSTs (Vig et al., 2009). The 3-IPMD was classified as an antioxidant upregulated by JA (Sasaki-Sekimoto et al., 2005). Yet it is present in larger amount in *cpm2* which lacks JA. Amongst the redox category, non-symbiotic hemoglobin2 (nSHb2) was the only protein reported to be less abundant in *cpm2* (Figure 8). Limited information is available about this class of protein or its probable function under abiotic stress, but it is known to be upregulated under hypoxia in plant roots (Riquelme and Hinrichsen, 2015). A positive correlation was established between nSHb2 and the amount of ω-3 α-linolenic acid, a precursor of JA, in the *Arabidopsis* seeds (Vigeolas et al., 2011). It cannot be said at this stage if the lack of JA in *cpm2* was a reason for reduced levels of nSHb2 under drought.

#### DAPs in energy-generating carbon/nitrogen catabolism indicates higher resource mobilization in *cpm2*

Glycolysis is a major metabolic pathway participating in carbohydrate metabolism, and drought stress triggers altered amino acid and sugar contents (Raorane et al., 2015b,a). In *cpm2*, three DAPs related to carbon catabolism were more abundant; triose phosphate isomerase (TPI), glyceraldehyde 3-phosphate dehydrogenase (G3PD) and pyruvate decarboxylase (PDC) (Figure 8). G3PD may serve as a direct link relating energy metabolism, membrane lipid–based signaling and control of a plant's growth in response to ROS and water stress (Guo et al., 2012). TPI is reported to participate in plant stress response and under drought stress, its expression is also induced in maize (Riccardi et al., 1998). Being the first and key participating enzyme during ethanolic fermentation, pyruvate decarboxylase (PDC) splits the main glycolytic pathway at pyruvate (Zabalza et al., 2009). Ethanolic fermentation is reported to occur not only under anaerobic conditions but also under stress conditions (Chen and Han, 2011). Similarly, some protein catabolism-related enzymes specific to proteasomes, such as, the threonine proteases T1-family peptidases, were more abundant in *cpm2* (Figure 8). Increased proteasome activity under stress is related to degradation of oxidized proteins, thus contributing to tolerance of oxidative stress (Kurepa et al., 2009). Protein degradation feeds into a favorable nitrogen status of the roots under drought (Kohli et al., 2012). A favorable nitrogen status of *cpm2* under drought was also supported by more abundance of Glutamine Synthetase (Figure 8). Serving as the first enzyme in the nitrogen assimilatory pathway, GS is known to regulate nitrogen metabolism and plant productivity (Lea and Miflin, 2011) and has been shown to be upregulated at the transcript and protein level under drought in rice (Raorane et al., 2015b,a). It is also reported that increased expression of GS enhances drought and salinity tolerance (Kalamaki et al., 2009).

#### DAPs-mediated changes in root cell walls suggests more active root development in *cpm2*

Roots quickly respond to changes in water potential and significantly alter morpho-physiological traits (Henry et al., 2012; Sandhu et al., 2016). Several DAPs such as a glycoside hydrolase (GH; family 17), *O*-methyl transferase (OMT), UDP-d-Glucose dehydrogenase, tubulin, and actin nucleation protein (Figure 8) suggested the ability of *cpm2* roots to stimulate cell wall adaptations during drought. GH17 endohydrolases cleave 1,3-d-glucosidic linkages linking β-1,3-glucans and therefore affect cell-wall assembly and rearrangement. GH17 endohydrolase activity has been linked with improved performance of rice plants under stress conditions such as, mechanical wounding or fungal stress (Jwa et al., 2006). The GH17 endohydrolase as a DAP was more abundant in *cpm2* (Figure 8). Similarly, an OMT was more abundant in *cpm2* (Figure 8). It is evident that OMTs may participate in several biotic and abiotic stress responses (Barakat et al., 2011). Being multifunctional enzymes, OMTs are reportedly known to play diverse role in various processes such as cell wall adaptation and catalyzing *O*-methylation of multiple secondary metabolites that are known participants during metabolic processes like plant growth, development, and environmental cues (Kim et al., 2010; Urbanowicz et al., 2012). A UDP-D-glucose dehydrogenase (UDPGDH) was also present in larger amount in *cpm2* (Figure 8). UDPGDH oxidizes UDP-D-glucose (UDP-Glc) to UDP-D-glucuronate (UDP-GlcA) the known precursor of UDP-xylose and UDP-arabinose, the precursors of arabinoxylans, the major hemicellulosic polysaccharide in the graminaceous cell wall (Kärkönen, 2005). More UGPGDH in *cpm2* suggests increased biosynthesis of hemicelluloses and cell wall remodeling in response to stress (Kärkönen et al., 2005). Yoshimura et al. (2008) also reported accumulation of UDPGDH in watermelon roots during drought stress.

Adaptive growth of *cpm2* roots under drought relates well to higher amounts of enzymes engaged in the formation of cell walls. Microtubules are an essential component of the cell's cytoskeleton, and tubulins are a crucial component of these microtubules that are functional during numerous cellular processes, ranging from cell division to intracellular transport inside eukaryotic organisms (Zhao et al., 2014) and determination of the orientation of cellulose fibrils in the cell wall (Schmit and Nick, 2008). β-Tubulin-1 was reported as more abundant in *cpm2* (Figure 8). Likewise, an actin nucleation protein, which acts in cell elongation, cell shape maintenance, and polarized growth of root hair (Ramachandran et al., 2000) was also more abundant in *cpm2* (Figure 8). Upregulation of the microtubule components under drought in rice roots has been reported earlier (Raorane et al., 2015b,a).

#### DAPs in phenylpropanoid pathway increased cell wall modification in *cpm2*

Water deficiency modulates the expression of lignin biosynthesis genes (Santos et al., 2015). Of the nine identified DAPs related to cell wall biosynthesis and remodeling, six encompass lignin biosynthesis and five of these belong to the phenylpropanoid biosynthetic pathway (Table 1). Such DAPs more abundant in *cpm2* were OsPAL1, Os4CL3, OsCAD4, OsCOMT1, and OsCCoAOMT (Figure 8, Figure S5), and these are crucial for lignin biosynthesis in rice plants (Gui et al., 2011; Hirano et al., 2012; Koshiba et al., 2013; Yoon et al., 2015; Wang et al., 2017). Transcript analysis of these genes revealed some interesting trends. *OsPAL1* increased in both genotypes under moderate stress, but under severe stress it was similar in wild type and *cpm2* (Figure S8A). The *Os4CL3* transcript was more abundant in *cpm2* under moderate and severe drought (Figure S8B) while the *OsCOMT1* transcript was similarly upregulated in *cpm2* under moderate stress but was not significantly different under severe stress (Figure S8C). Taken together, the transcript and protein abundance data in the phenylpropanoid pathway suggested an increase in the biosynthesis of phenolics and/or lignin (Vincent et al., 2005; Fan et al., 2006; Phimchan et al., 2014) in *cpm2*.

Since coumarates are also known to be incorporated into the lignin structure, increased lignin in *cpm2* suggested increased coumarates. The level of esterified *p*-coumarates was higher under drought in the WT but not in *cpm2* (Figure 7A). Phloroglucinol staining for lignifications also showed increase in lignin in the WT root section but not in *cpm2* (Figure S9). To resolve the discrepancy in *cpm2* between the potentially increased biosynthesis of phenolics/lignin but relatively lesser lignin content visualized through phloroglucinol, quantitative estimation of the changes in the lignin polymer composition (monolignol ratios and linkage types within the polymer) will be conducted in future, because phloroglucinol best stains cinnamaldehydes (Adler, 1977) while the extent of lignin crosslinking can affect visualization by phloroglucinol (Pomar et al., 2002). The increase of the elastic modulus but reduced plastic extensibility of the cell wall under drought (Blum, 2011) is caused in part by augmented cross-linking of arabinoxylans to each other and lignin via free-radical-induced oxidative coupling of phenolics like lignin monomers and ferulates (Fan and Neumann, 2004; Fan et al., 2006).

*p-*Coumaric acid is also found ester-linked to arabinoxylans just like ferulic acid (Mueller-Harvey et al., 1986). No significant difference in the arabinose/xylose ratio was found in the *cpm2* roots' cell wall material under drought in contrast to WT, which had a lower arabinose/xylose ratio in the drought-stressed roots compared to the control (Figure S10). The arabinose/xylose ratio is an indicator of the degree of substitution of the arabinoxylan polymer backbone, which has been shown to be reduced in drought-stressed plants (Rakszegi et al., 2014). Contrary to our expectations based on the protein biosynthesis data, drought-stressed *cpm2* roots showed reduced biosynthesis of both ester-linked ferulic acid monomers (Figure 7B) and the oligoferulates (dimers and trimers) involved in cell-wall crosslinking compared to the control (Figure S6). Drought stress also resulted in fewer diferulates in the WT roots compared to the control (Figure S6B). Both osmotic stress and ABA have been previously shown to reduce ester-linked ferulate and diferulate content in wheat coleoptiles (Wakabayashi et al., 1997a,b). The results indicate that rice roots respond to drought stress by loosening cross-links in the cell wall, to become more flexible. However, JA might inhibit this response, as WT roots show a weaker decrease in those crosslinking metabolites under drought stress.

Discrepancies in the results expected for the *cpm2* cell walls from more abundant DAPs for phenylpropanoid and ferulate pathways could potentially be ascribed to alternate uses of the secondary metabolite products of the enzymes involved. For example, the phenylpropanoid pathway involving chorismate and shikimate could be directed to salicylic acid (SA) synthesis, which like JA is perhaps equally but antagonistically involved in drought response (Pedranzani and Vigliocco, 2017).

#### Lower abundance of DAPs for aquaporins in *cpm2* could influence root water status positively

Plasma membrane intrinsic proteins (PIP), a subfamily of aquaporins comprising two subgroups of PIP1 and PIP2 could also influence root water status. PIP2 proteins could be attributed for enhanced water channel activity (Chaumont et al., 2000). In the present study, OsPIP1 was less abundant in *cpm2* (Figure 8). Reduced expression of some of the PIP genes could lead to inhibition of water loss by preventing backflow of water to dried soil (Afzal et al., 2016). Moreover, down-regulation of PIP genes transcription was also reported in the roots of tobacco (Mahdieh et al., 2008) and also in the twigs and roots of olive plants (Secchi et al., 2007).

### Impaired AOC function useful for drought tolerance

Drought stress apparently upregulates the JA biosynthetic pathway as evident at the transcript and protein level in our study and in earlier reports in rice (Du et al., 2013). In the root proteome analysis AOC was unique and highly abundant under drought in the WT (Figure 6A). Moreover, we also found 12-OXOPHYTODIENOATE REDUCTASE (OPR7), another protein downstream of AOC in the JA biosynthesis pathway, was more abundant under drought in *cpm2* roots (Figure 6A). However, when comparing OPDA, JA, and JA-Ile levels between WT and *cpm2* shoots and roots under control, moderate and severe stress, minor differences were found, except in the OPDA levels in WT shoots during severe stress (Figures S11A–F). Even though the amounts detected for these hormones under our specific experimental conditions were rather low.

These results raise two important questions. First, how can upregulation of JA synthesis genes under drought be reconciled with its proposed role as a negative regulator of drought tolerance?, the latter ascertained from morpho-physiological responses and root proteome indicators in *cpm2* that lacks JA. Possibly, increase in JA content under drought upregulates some JA responsive genes (JRG) that are negative regulators of drought tolerance. Hence lack of JA in *cpm2* does not upregulate the negative regulators of drought tolerance. For example, one of the JRGs, *chlorophyllase 1* (*CLH1*) is a senescence associated gene (Sasaki et al., 2001) the upregulation of which may not be useful. JA induced natural senescence in maize has been reported (Yan et al., 2012). Additionally, the complex crosstalk of JA with other plant hormones such as ABA, salicylic acid (SA) and ethylene may dictate its final effect. For example, another JRG highlighted by Sasaki et al. (2001) is a SA-glucosyltransferase, which can inhibit SA signaling while SA is known to be important in abiotic stress tolerance (Pedranzani and Vigliocco, 2017). Also, recently Liu and Avramova (2016) reported that JA was not able to induce any of the dehydration response genes tested but it potentiated ABA-dependent genes. Thus JA crosstalk to other hormones may underpin the drought response rather than a direct relationship between its upregulation and action. Second, under the lack of AOC protein and hence the suspected lack of 12-OPDA, the substrate for OPR7, how *OPR7* can be upregulated? Potentially the transcription and translation of downstream *OPR7* gene was not dependent on the product of AOC activity (12-OPDA). Similar to our results in Figure S11, detectable amounts of OPDA and JA remained in the maize single mutants for OPR7 as observed by Yan et al. (2012) who speculated other members of the OPR gene family to be responsible for low but measurable amounts of JA in the *opr7* mutant. However, similar results of detectable OPDA and JA synthesis in the mutants of two different genes for JA synthesis, in two different crops, present an intriguing scenario and suggest unexplored cryptic regulation of JA biosynthesis. It must be noted that OPDA and JA content in the WT roots under drought was as low as that of the *cpm2* (Figures S11D,E). This suggested that the comparatively decreased contents of OPDA and JA in *cpm2* under the control conditions had some effect rather than the drought-mediated decrease *per se*. Finally, since hormone levels are transient it is possible to have missed the critical time points during which alterations could be detected. Nevertheless, from the proteomic evidence, it can be speculated that in the WT roots, owing to the abundance of AOC but scarcity of the downstream OPR7, OPDA is accumulated. This speculation is also in agreement with the findings of Savchenko et al. (2014). Moreover using *cpm2* and corresponding WT for a study on salinity stress, Hazman et al. (2015) reported that an increase in OPDA content in WT was perceived as a damage signal. We observed similar increase in the OPDA amounts at least in the shoots of the WT during severe stress condition (Figure S11A), but in order to further validate this finding at the metabolite level in drought stressed roots, further quantification of jasmonates must be conducted at the more precise and early time point after onset of stress.

### Conclusions and the proposed role of JA in response to drought

In *cpm2*, the response to drought stress at the initiation of water deficit condition was much faster than in the WT, as also evident from its lower stomatal conductance. Overall, the mutant displayed a better fitness to combat drought stress conditions through effective activation of stress-preventing mechanisms that further led to its better sustenance under drought.

In summary, in comparison to the WT, *cpm2* exhibited:

1. morpho-physiological adaptations to drought through lower stomatal conductance (at the onset of stress), higher ABA levels and higher WUE.
2. Better root system under control and drought conditions as revealed by WinRHIZO based measurements.
3. Comparative root proteome analysis supported a better status of *cpm2* roots in terms of more efficient ROS scavenging, more abundance of cell wall-modifying, energy-generating, and water-saving enzymes/proteins.
4. Transcript and protein abundance data in the phenylpropanoid pathway suggested an increase in the biosynthesis of phenolics and/or lignin in *cpm2*. Moreover, cell wall metabolite analysis revealed a unique profile for *cpm2* under drought stress. However, in the future quantitative measurements of the lignin polymer composition (monolignol ratios and linkage types within the polymer) will be necessary to further validate these observations.
5. In the WT shoots, OPDA levels were found to be higher under severe drought. Similarly, in the WT roots, based on proteomic results OPDA is speculated to be accumulated due to high abundance of AOC and scarcity of OPR7. These observations are in agreement with the previous findings of Savchenko et al. (2014) and Hazman et al. (2015), however this finding for drought also needs to be further validated at metabolite level in the roots wherein, more early time-point samples need to be collected after the start of stress treatment—to further detect immediate and direct changes in these hormones. Additionally, potential hormone cross-talk was implicated through our study, which warrants further research, namely that ABA may need JA for stomatal closure and that JA effects may be modulated through the involvement of SA.

All of the aforesaid attributes allowed *cpm2* to optimize its growth in response to a water deficit condition and to further withstand a prolonged drought stress. Presently, it is not possible to define the exact mechanisms controlling better drought responses in *cpm2*. However, based on the integration of root proteome, metabolite and morpho-physiological data, we can rather propose the key molecular players involved in the better sustenance of *cpm2* during drought. We believe that the results presented in this study are a key in further advancements toward understanding the precise role of JA during drought stress tolerance and also the molecular mechanisms involved. Thus, we conclude that drought response of *cpm2* implicated JA as a negative regulator of root growth and overall drought tolerance.

## Author contributions

RD performed the morpho-physiological characterization experiments, generated all plant sampling material, prepared RNA and protein samples, performed TMT labeling experiments, analyzed and interpreted the morpho-physiological, cell wall metabolite and root proteome results, and wrote the manuscript; MLR helped in sampling plant materials, performed transcript analysis of *AOC* and *OPR7*, interpreted the proteome and metabolite data and participated in writing the manuscript. AT performed the LC-MS/MS and bioinformatics analysis for the root proteomics data. PP performed the qRT-PCR analysis of genes related to phenylpropanoid pathway. RS and MB performed the cell wall metabolite analysis and its interpretation, respectively. VS performed the root histological studies. BH performed the plant hormone measurements and participated in manuscript writing. AH designed and supervised the physiological experiments and participated in interpreting the morpho-physiological data. AK and MR together conceptualized and supervised the whole study as well as participated in writing the manuscript.

### Conflict of interest statement

The authors declare that the research was conducted in the absence of any commercial or financial relationships that could be construed as a potential conflict of interest.
