# Corrigendum: Physiological and Proteomic Analysis of the Rice Mutant *cpm2* Suggests a Negative Regulatory Role of Jasmonic Acid in Drought Tolerance

**DOI:** 10.3389/fpls.2018.00465

**Published:** 2018-04-10

**Authors:** Rohit Dhakarey, Manish L. Raorane, Achim Treumann, Preshobha K. Peethambaran, Rachel R. Schendel, Vaidurya P. Sahi, Bettina Hause, Mirko Bunzel, Amelia Henry, Ajay Kohli, Michael Riemann

**Affiliations:** ^1^Molecular Cell Biology, Institute of Botany, Karlsruhe Institute of Technology, Karlsruhe, Germany; ^2^International Rice Research Institute, Los Baños, Philippines; ^3^Newcastle University Protein and Proteome Analysis, Newcastle University, Newcastle Upon Tyne, United Kingdom; ^4^Department of Food Chemistry and Phytochemistry, Institute of Applied Biosciences, Karlsruhe Institute of Technology, Karlsruhe, Germany; ^5^Cell and Metabolic Biology, Leibniz Institute of Plant Biochemistry, Halle, Germany

**Keywords:** jasmonates, rice, drought, root, proteomics, phytohormones, cross-talk

In the original article, we neglected to include the partial funding provided to RD by German Federal Ministry for Research and Education and the Egyptian Science and Technology Development Fund. Therefore, the following statement should be added to the acknowledgment:

“This work has been supported by funds to RD by the German Federal Ministry for Research and Education and Egyptian Science and Technology Development Fund (01DH14013).”

The authors apologize for this error and state that this does not change the scientific conclusions of the article in any way.

The original article has been updated.

## Conflict of interest statement

The authors declare that the research was conducted in the absence of any commercial or financial relationships that could be construed as a potential conflict of interest.

