# Supplementary material for: Physiological and Proteomic Analysis of the Rice Mutant cpm2 Suggests a Negative Regulatory Role of Jasmonic Acid in Drought Tolerance
Source: Front Plant Sci. 2017 Nov 10;8:1903. doi: 10.3389/fpls.2017.01903 (PMC5715382; doi:10.3389/fpls.2017.01903)
Supplement: Supplementary file 1 [file Table1.DOCX]

**Table S1. The sequences of gene specific forward and reverse primers used for the qRT-PCR analysis**

| **Primer Name** | **Gene name** | **Accession number** | **Forward Primer (5’-3’)** | **Reverse Primer (5’-3’)** |
| --- | --- | --- | --- | --- |
| OsCyclophilin-2 | Cyclophilin-2 | LOC_Os02g02890 | GTGGTGTTAGTCTTTTTATGAGTTCGT | ACCAAACCATGGGCGATCT |
| OsAOC | Allene Oxide Cyclase | LOC_Os03g32314 | TGCCTCAACAACTTCACCAACTA | CACATGCCGCAATTAACACTAAA |
| OsOPR7 | 12-Oxophytodienoate Reductase-7 | LOC_Os08g35740 | CTCAACCACCGGTTTCCTCA | TCCATGCATCAGTCTGCTCT |
| OsPAL1 | Phenylalanine-ammonia-lyase-1 | LOC_Os02g41630 | AAGGTGTTCCTCGGCATCAG | GGCAATGGCGATGGGATCTT |
| Os4CL3 | 4-coumarate-CoA ligase-3 | LOC_Os02g08100 | CTCACCCGGAGATCAAGGAC | CCTCGGTGATTTCTGAGCCT |
| OsCOMT1 | Caffein-o-methyltransferase-1 | LOC_Os08g38900 | TCATCACGGACAAGCACCAG | GACACCCACCTCGATTGTCC |
