# Supplementary material for: Physiological and Proteomic Analysis of the Rice Mutant cpm2 Suggests a Negative Regulatory Role of Jasmonic Acid in Drought Tolerance
Source: Front Plant Sci. 2017 Nov 10;8:1903. doi: 10.3389/fpls.2017.01903 (PMC5715382; doi:10.3389/fpls.2017.01903)
Supplement: Supplementary file 2 [file Presentation1.pdf]

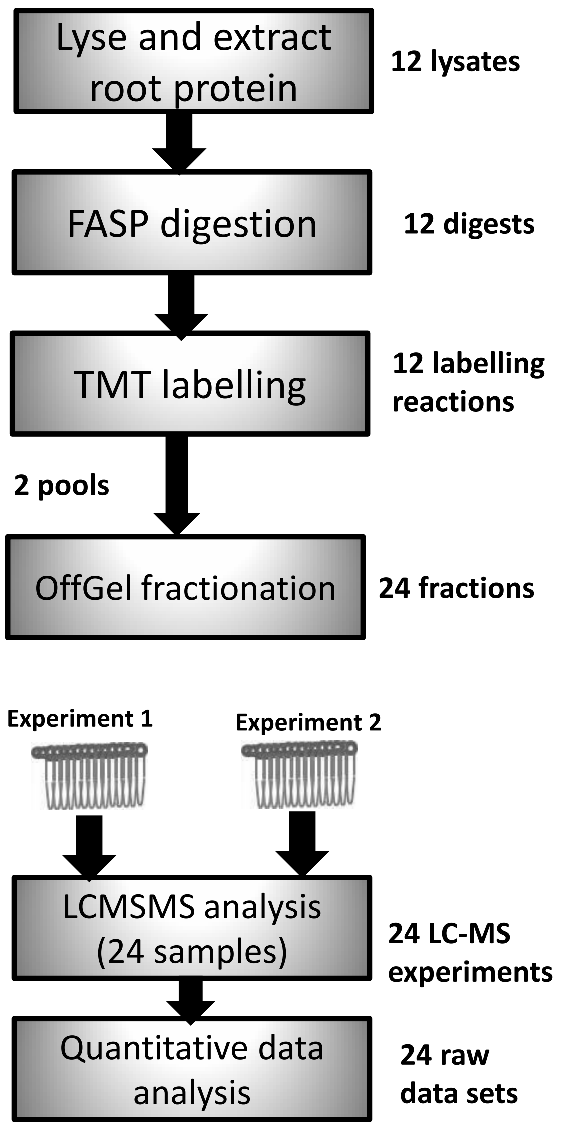

Figure S1. **Strategy for TMT (Tandem Mass Tag) based comparative proteomics of *cpm2* and corresponding wild type (WT).** Total protein extraction was performed on four week old root samples which were collected from drought stressed and control WT and *cpm2* plants. Total protein extract was digested with trypsin and the subsequent peptide mixture was labeled with the TMT reagent as per the manufacturer protocol. Pooled peptides were fractioned using the reversed-phase HPLC system, then individual fractions were analyzed using LC-MS/MS. MS raw data were processed using the SWISSPROT protein database. Identified proteins with one or more than one peptide with MASCOT scores greater than 40 were immediately accepted. Single peptides with MASCOT scores less than 40 were deleted from the analysis to avoid false positives. The MSU TIGR v7.0 locus identifiers of the remaining proteins were retrieved using the ID mapping tool in UniProtKB (www.uniprot.org) for input into MapMan.

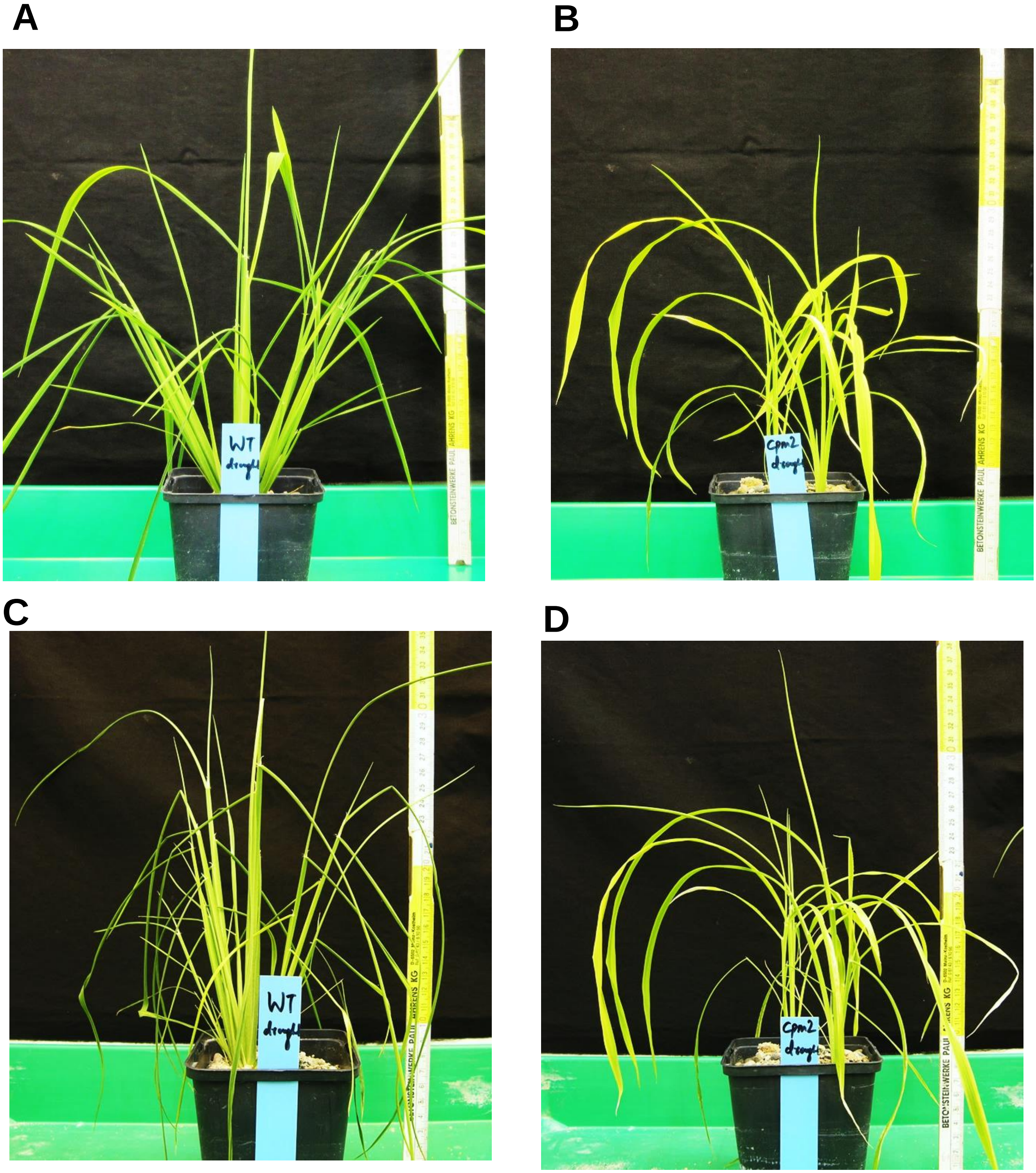

Figure S2. Phenotypic changes observed in rice seedlings after exposure of drought stress. Two days after exposure of drought stress on A) wild type (WT), and B) *cpm2*. Four days exposure of drought stress on C) WT, and D) *cpm2*. Four week old seedlings of WT and *cpm2* were subjected to drought stress and photographed subsequently after first appearance of drought stress symptoms.

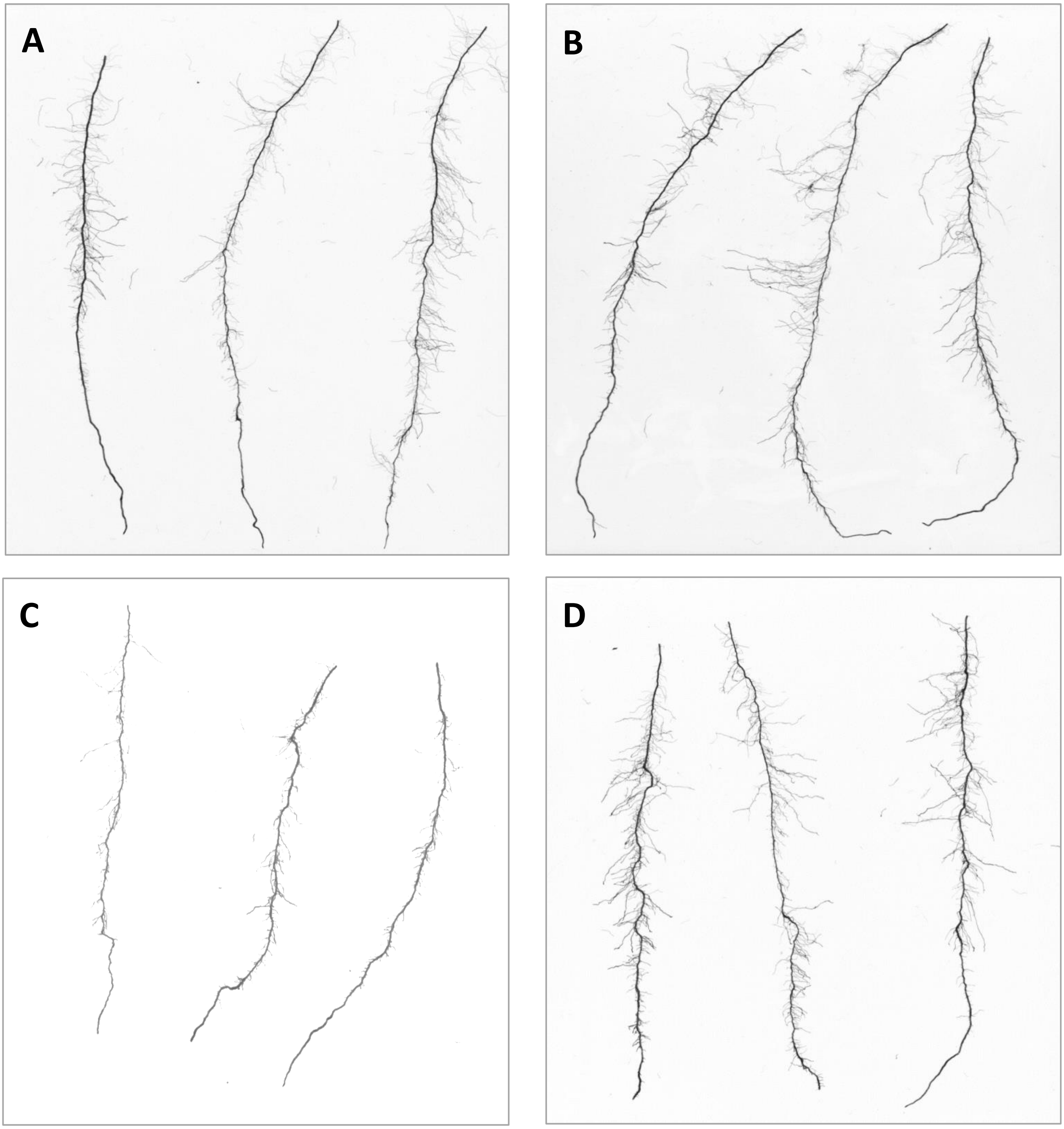

Figure S3. Images of three nodal roots from wild type (WT) and *cpm2*. Prior to scanning of roots, plants of two genotypes were grown in soils at different moisture levels. Under control conditions, significant visual differences were exhibited by B**)** *cpm2* over A) WT. Under moderate drought too, D) *cpm2* exhibited visually more root branches and thus a better-developed root system than the C) WT.

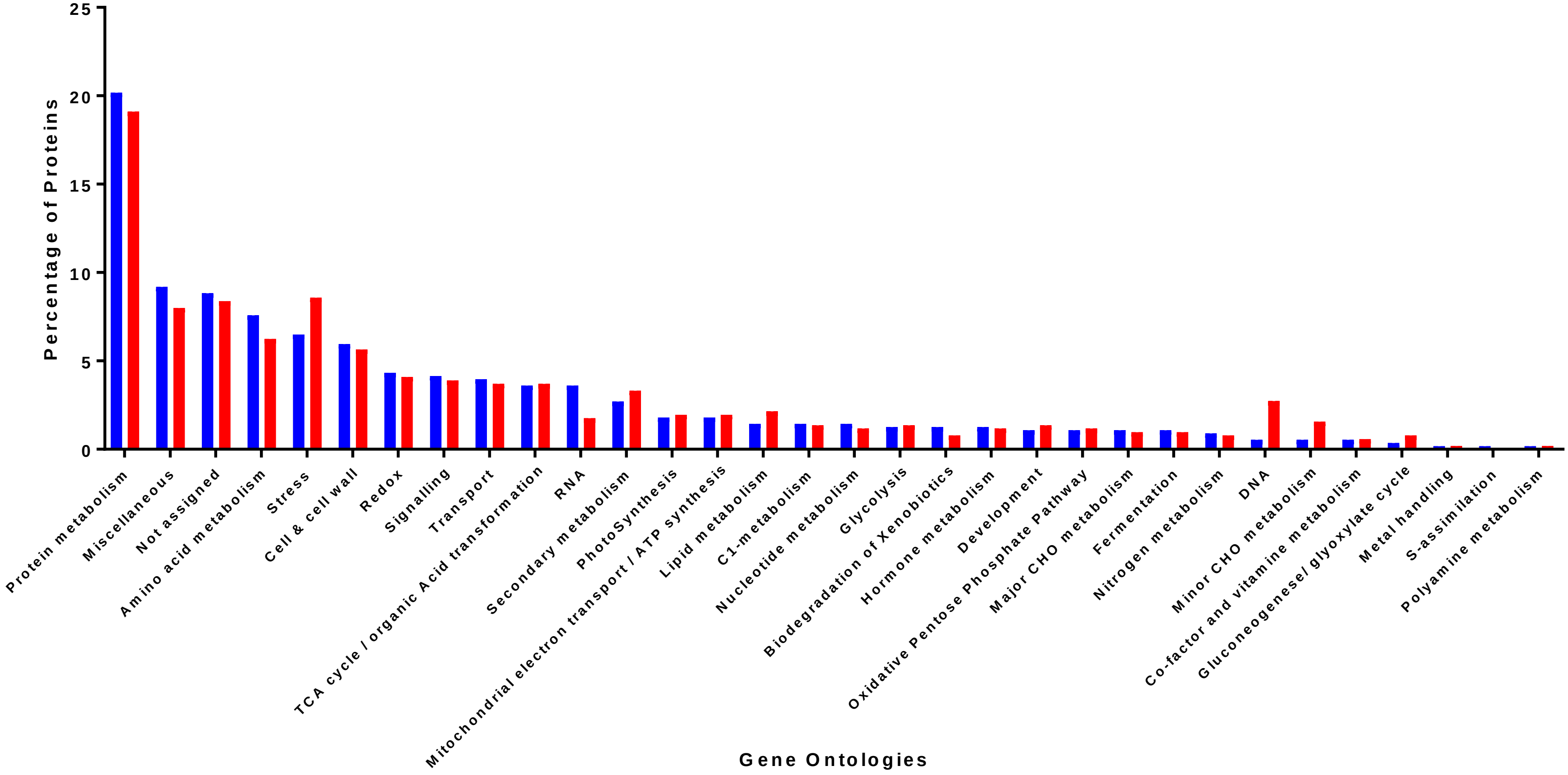

Figure S4. Overview of the percentage of proteins mapped onto MapMan based gene ontologies in the wild type (WT) and *cpm2* roots after TMT (Tandem Mass Tag) analysis. Proteins identified after TMT analysis were classified into 32 functional categories according to MapMan ontology. WT and *cpm2* are represented by blue and red bars, respectively.

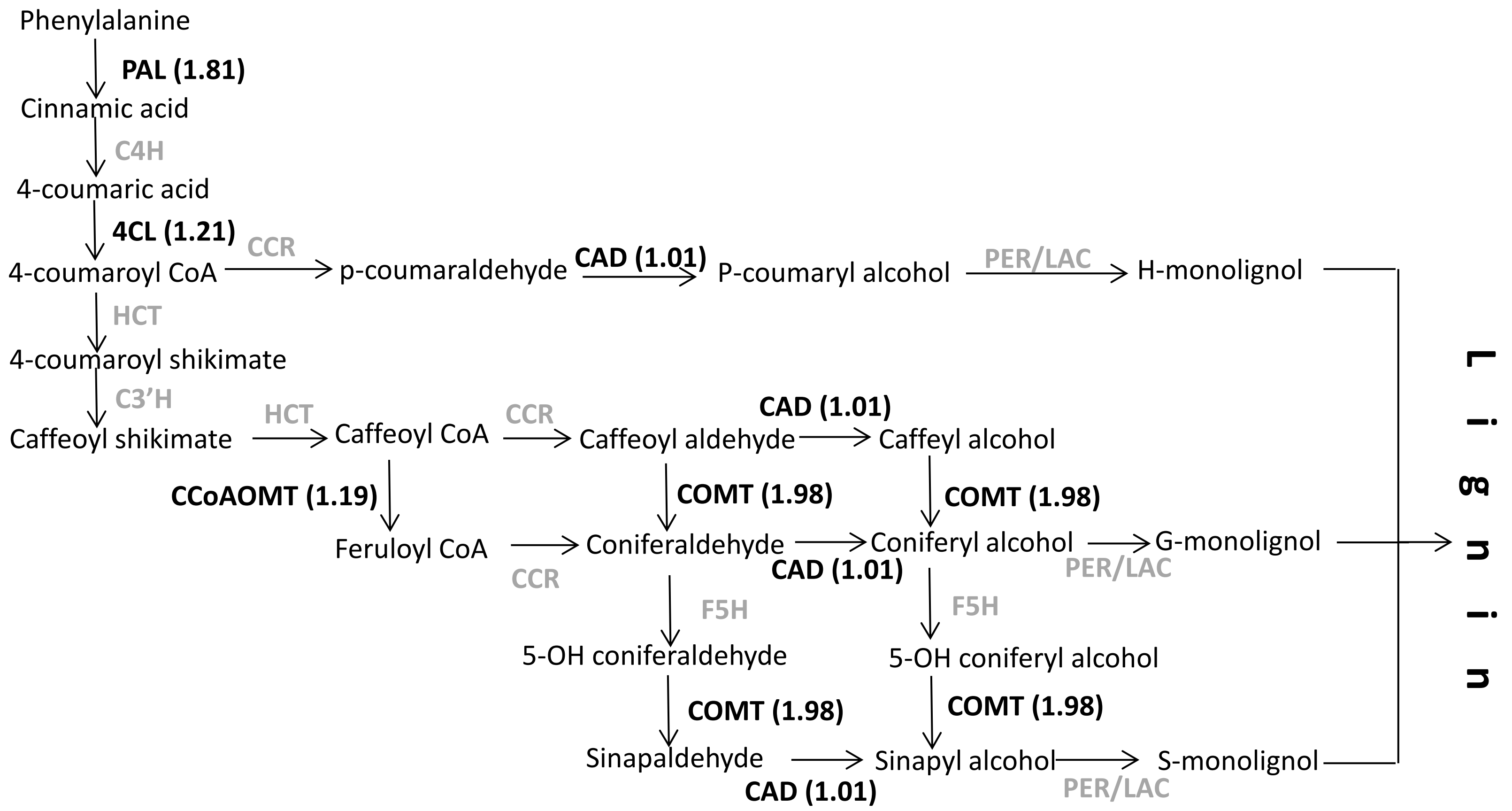

Figure S5. Proteins involved in phenylpropanoid pathway were uniquely abundant in *cpm2* roots. Proteins involved are unique and higlighted in black for *cpm2*, with their log ratios expressed as stress/control namely PAL, phenylalanine ammonia lyase; 4CL, 4-coumarate:coenzyme A ligase; COMT, caffeic acid O-methyltransferase; CAD, cinnamyl alcohol dehydrogenase; CCoAOMT, caffeoyl-CoA O-methyltransferase. The log ratios (stress/control) were obtained after TMT based high-throughput comparative proteome analysis performed on rice roots of *cpm2* and corresponding WT grown under greenhouse conditions.

Other abbreviations **:** C4H, cinnamate 4-hydroxylase; HCT, hydroxycinnamoyl-coenzyme-A-shikimate:quinate hydroxycinnamoyl-transferase; C3'H, p-coumaroyl shikimate 3'-hydroxylase; CCR, cinnamoyl CoA reductase; F5H, ferulate 5-hydroxylase; PER, peroxidase; LAC, laccase; Figure modified from Zhao and Dixon, 2011

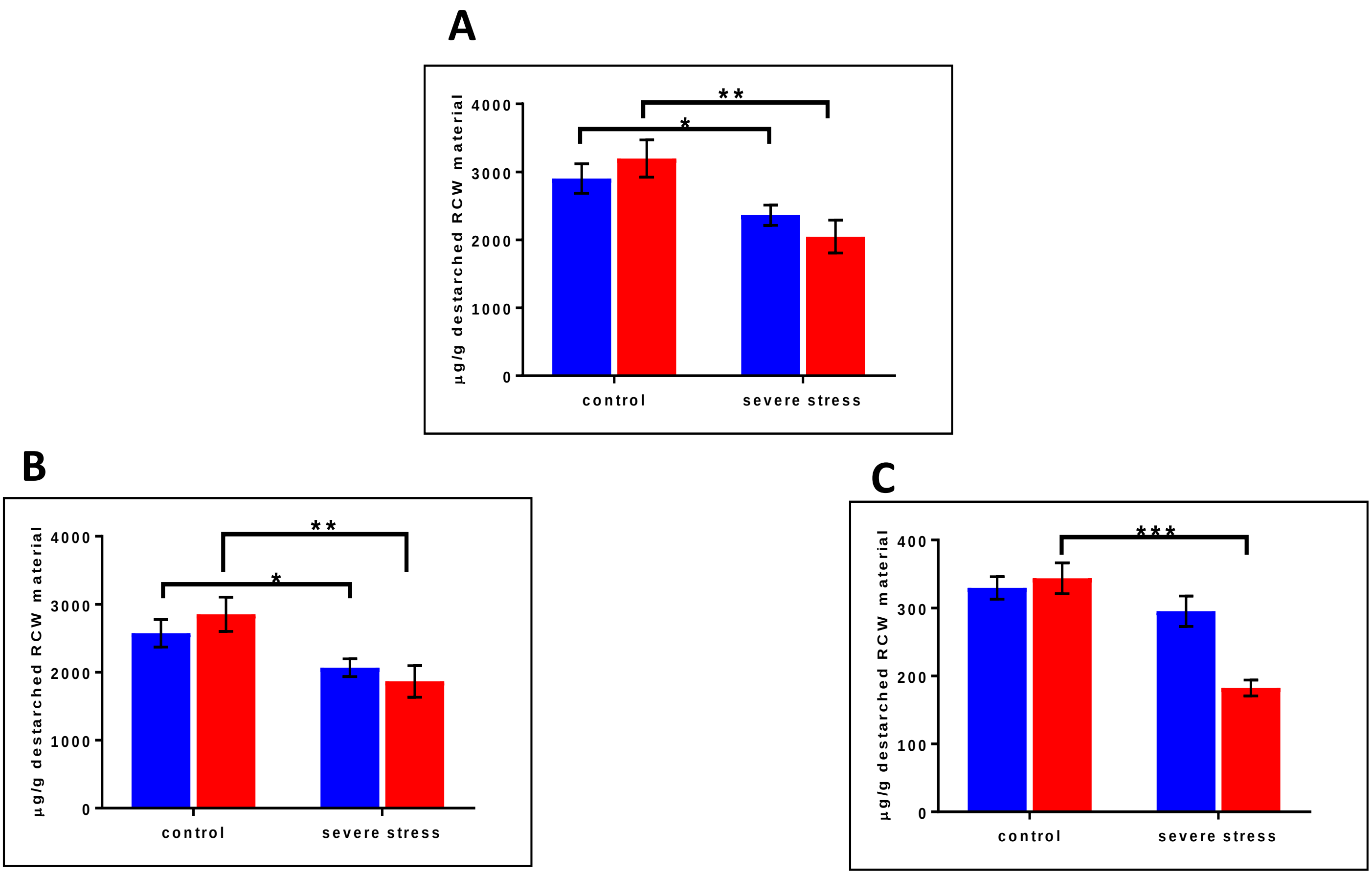

Figure S6. Changes in metabolic levels of ester-linked A) total oligoferulates B) di-ferulates and C) tri-ferulates in wild type (WT) and *cpm2* destarched root cell wall (RCW) material under control and severe drought stress conditions. Three-week-old WT and *cpm2* seedlings were either kept well-watered, and their roots were sampled at day 22, or watering was stopped to initiate severe stress condition. Root samples were harvested in control and severe stress condition. Ester-linked oligoferulates (dimers and trimers) were quantified via LC-MS/MS. WT and *cpm2* are represented by blue and red bars, respectively. Data represent mean value ± standard error (SE); n=5. Stars (*, ** & ***) denote statistical significance ($P \leq 0.05$, $P \leq 0.01$ & $P \leq 0.001$), respectively, between the two genotypes in a Student's *t*-test.

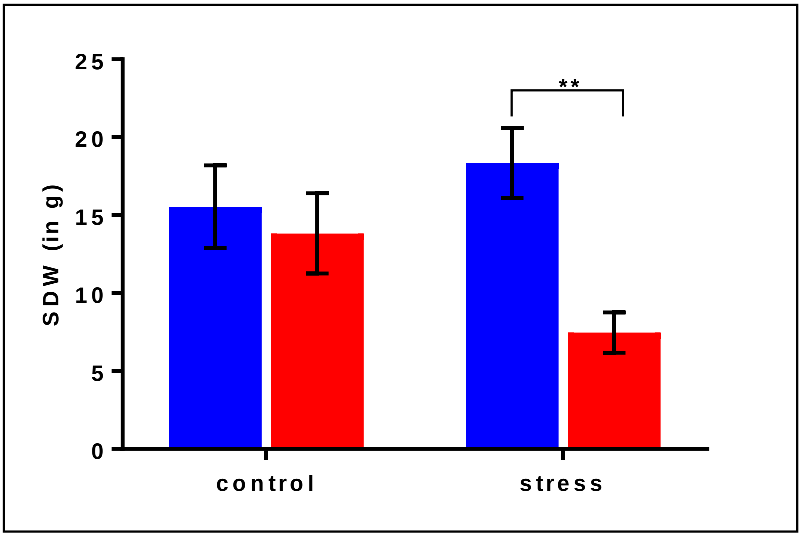

Figure S7. Shoot Dry Weight (SDW) of *cpm2* and wild type (WT) under control and drought stress. WT & *cpm2* plants were subjected to drought after 32 days of sowing by withholding water. At the end of water use efficiency (WUE) experiment, shoot tissue was extracted and kept at 70 °C for 96 hours, after which shoot dry weight was measured using a weighing balance. Significant differences were observed across the treatments. Data represent mean values ± standard error (SE); n=5. WT and *cpm2* are represented by blue and red bars, respectively. Stars (**) denote statistical significance (P≤0.01), between the two genotypes in a student's *t*-test.

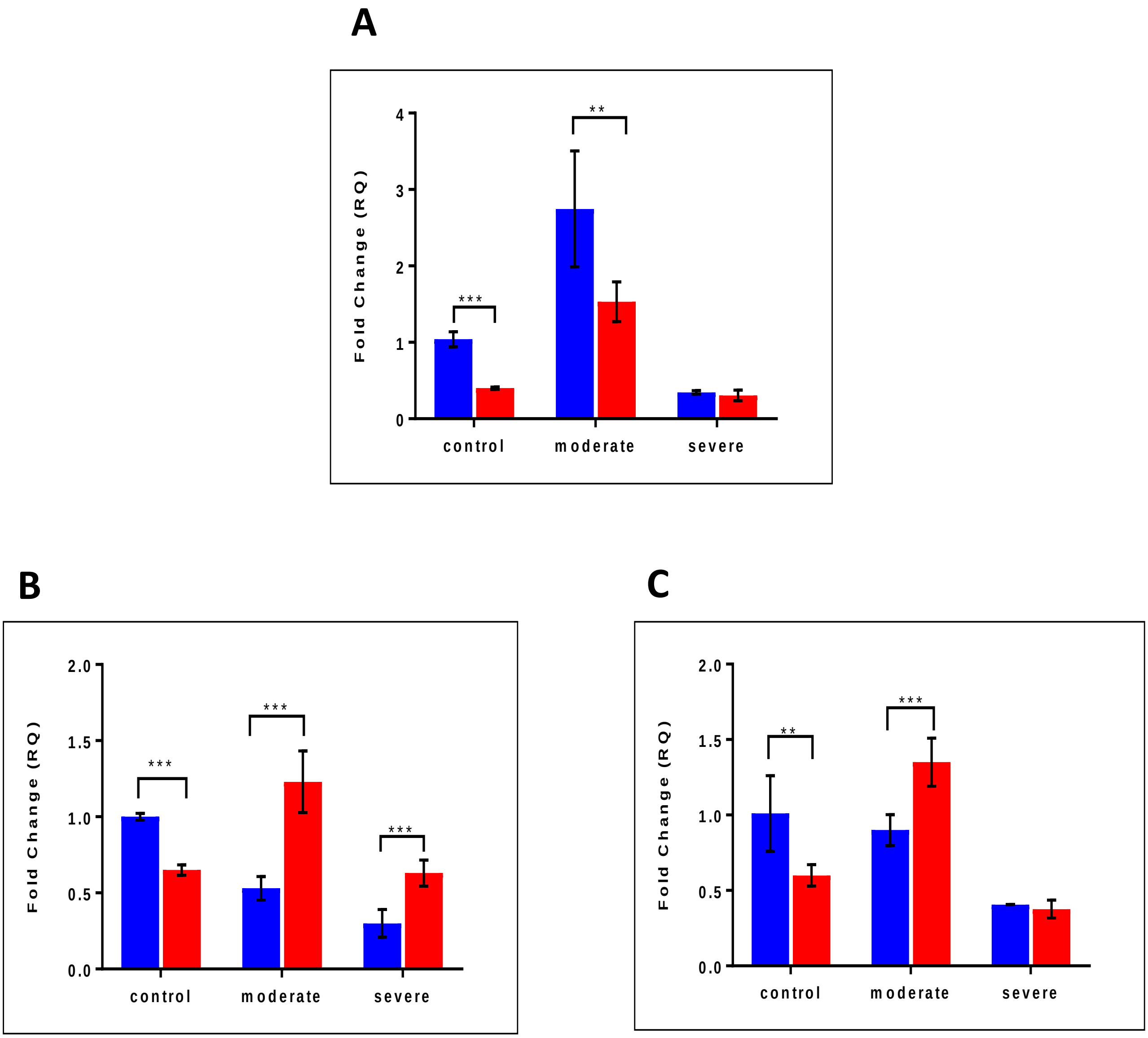

Figure S8. Alterations in transcript abundance of A) *OsPAL1* (LOC_Os02g41630) B) *Os4CL3* (LOC_Os02g08100) C) *OsCOMT1* (LOC_Os08g38900) in wild type (WT) and *cpm2* under control, moderate and severe drought stress conditions. Three-week-old WT and *cpm2* seedlings were either kept well-watered, and their roots were sampled at day 22, or watering was stopped to initiate moderate and severe stress conditions. Root samples were harvested in control, moderate and severe stress condition, respectively. WT and *cpm2* are represented by blue and red bars, respectively. Data represent mean value ± standard error (SE); n=5. Stars (** and ***) denote statistical significance ($P \leq 0.01$ and $P \leq 0.001$), respectively, between the two genotypes in a Student's *t*-test.

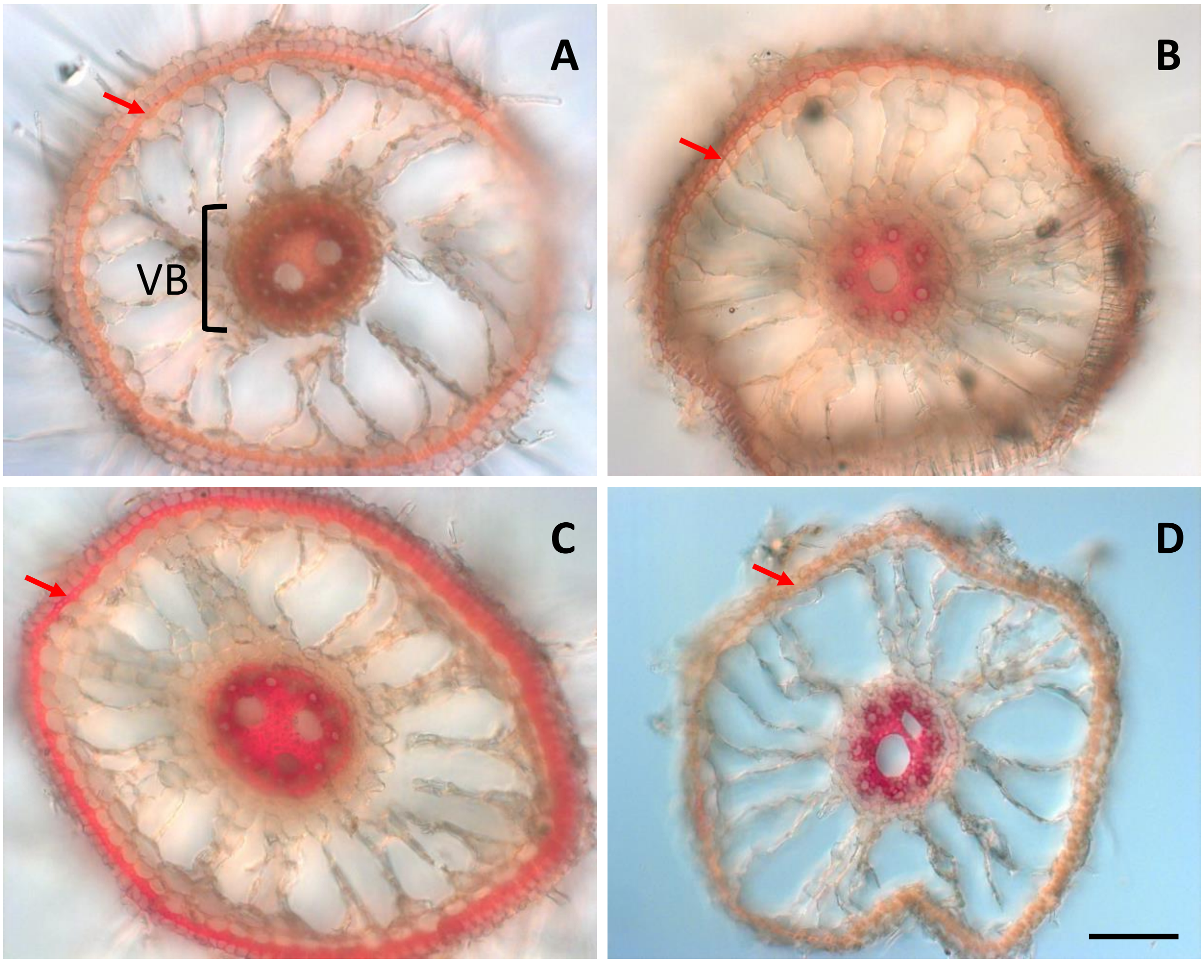

Figure S9. Differences in lignification between wild type (WT) and *cpm2* plants grown under control and severe drought conditions. Lignification of sclerenchyma and vascular bundle (VB) in A) WT control is visibly less than C) WT under drought. Differences in lignification were not significant between B) *cpm2* control and D) *cpm2* under drought. The red arrows point at sclerenchyma. VB: Vascular bundle. All images are in same scale and bar in D represents 100µm.

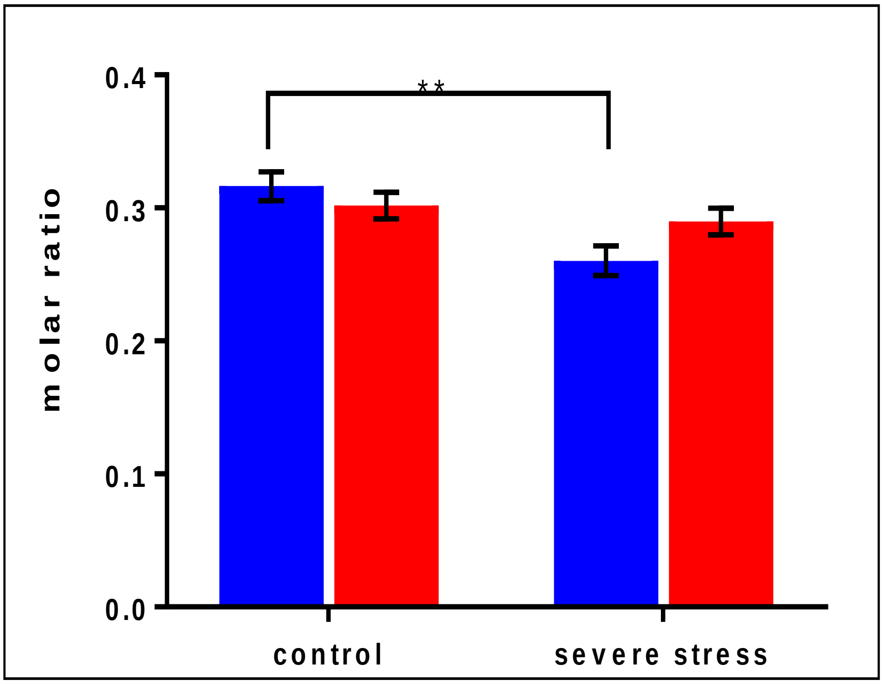

Figure S10. Arabinose to Xylose ratio (Ara/Xyl) under control and severe stress condition in the WT and *cpm2* roots. Ara/Xyl is an indicator of degree of branching on the arabinoxylan polymer backbone. The ratio was unchanged in *cpm2* under drought as compared to control. Wild type (WT) and *cpm2* are represented by blue and red bars, respectively. Data represent mean value ± standard error (SE); n=5. Stars (**) denote statistical significance ($P \leq 0.01$), respectively, between the two genotypes in a Student's *t*-test.

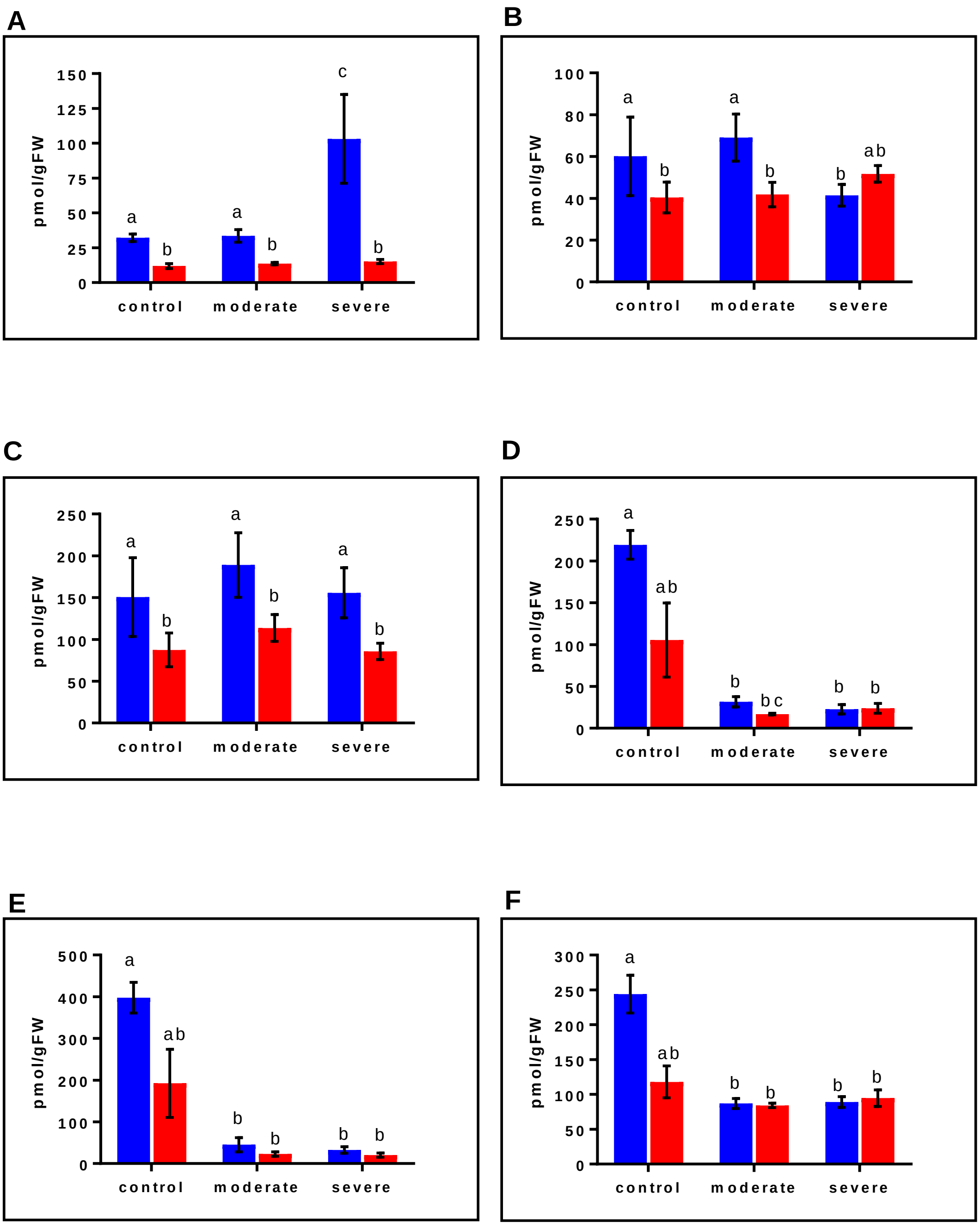

Figure S11. Levels of jasmonates in WT and *cpm2* under control and drought stress conditions. Levels of (A) 12-*cis*-oxophytodienoic acid (OPDA), (B) jasmonic acid (JA), and (C) JA–isoleucine (JA-Ile), in shoots. Levels of (D) 12-*cis*oxophytodienoic acid (OPDA), (E) jasmonic acid (JA), and (F) JA–isoleucine (JA-Ile), in roots. WT and *cpm2* are represented by blue and red bars, respectively. Data represent mean value ± standard error (SE); n=5. Means followed by different letters among treatments are significantly different, according to ANOVA single-factor test with respect to Tukey's Honestly Significant Difference (HSD) test (P<0.05). Three-week-old wild type (WT) and *cpm2* seedlings were either kept well-watered, and their shoots and roots were sampled at day 22, or watering was stopped to initiate moderate and severe stress conditions. Root and shoot samples were harvested in control, moderate and severe stress condition, respectively.
